# Elevated SLC3A2 associated with poor prognosis and enhanced malignancy in gliomas

**DOI:** 10.1038/s41598-024-66484-1

**Published:** 2024-07-09

**Authors:** Yuheng Xu, Wanqi Weng, Yuhao Weng, Danmin Chen, Ziwen Zheng, Zexian Fan, Chengxiang Peng, Yuanyi Xiong, Xiao Pang, Guobin Cao, Yezhong Wang, Quan Mo, Zhaotao Wang, Shizhen Zhang

**Affiliations:** 1 Department of Neurosurgery, Institute of Neuroscience, The Second Affiliated Hospital of Guangzhou Medical University, Guangzhou, 510260 China; 2 Department of Anesthesiology, The Second Clinical College of Guangzhou Medical University, Guangzhou, 510182 China; 3 Department of Neurosurgery, Huaiji County People’s Hospital, Zhaoqing, 526400 China

**Keywords:** Disulfidptosis, SLC3A2, Glioma, Immune infiltration, Prognosis, Biomarkers, Predictive markers, Prognostic markers, CNS cancer

## Abstract

The role of SLC3A2, a gene implicated in disulfidptosis, has not been characterized in gliomas. This study aims to clarify the prognostic value of SLC3A2 and its influence on glioma. We evaluated the expression of SLC3A2 and its prognostic importance in gliomas using publicly accessible databases and our clinical glioma samples and with reliance on Meta and Cox regression analysis approaches. Functional enrichment analyses were performed to explore SLC3A2's function. Immune infiltration was evaluated using CIBERSORT, ssGSEA, and single-cell sequencing data. Additionally, Tumor immune dysfunction and exclusion (TIDE) and epithelial-mesenchymal transition scores were determined. CCK8, colony formation, migration, and invasion assays were utilized in vitro, and an orthotopic glioma xenograft model was employed in vivo, to investigate the role of SLC3A2 in gliomas. Bioinformatics analyses indicated high SLC3A2 expression correlates with adverse clinicopathological features and poor patient prognosis. Upregulated SLC3A2 influenced the tumor microenvironment by altering immune cell infiltration, particularly of macrophages, and tumor migration and invasion. SLC3A2 expression positively correlated with immune therapy indicators, including immune checkpoints and TIDE. Elevated SLC3A2 was revealed as an independent risk element for poor glioma prognosis through Cox regression analyses. In vitro experiments showed that reduced SLC3A2 expression decreased cell proliferation, migration, and invasion. In vivo, knockdown of SLC3A2 led to a reduction in tumor volume and prolonged survival in tumor-bearing mice. Therefore, SLC3A2 is a prognostic biomarker and associated with immune infiltration in gliomas.

## Introduction

Gliomas represent the most common type of primary brain tumor, which are classified into low-grade gliomas (LGGs) and glioblastomas (GBMs). The highly aggressive and heterogeneous nature of gliomas makes them prone to recurrence and have a low 5-year survival rate^1–3^. Despite research advances, therapeutic alternatives are still restricted and survival has only marginally improved, necessitating the discovery of novel biomarkers for effective diagnosis and targeted treatment^4,5^.

Recent advancements in cancer pathophysiology have spotlighted the importance of metabolic reprogramming in cell survivability and proliferation. Among these, disulfidptosis, a cell death pathway precipitated by oxidative stress and characterized by the accumulation of disulfide bonds, results in a unique form of mortality known as 'disulfide stress'^6^. Professor Gan Boyi and colleagues have illuminated the role of SLC7A11 in this process, while subsequent CRISPR-Cas9 screenings have implicated its close partner, SLC3A2, as well^6,7^. SLC3A2, a component of the cystine/glutamate antiporter, has been characterized as a novel regulator of cellular antioxidant defenses and metabolic homeostasis^8,9^. SLC3A2's expression patterns across various cancers have been linked to poor patient outcomes, suggesting its role in oncogenic signaling pathways^10,11^. Elevated SLC3A2 levels have been associated with enhanced tumor growth, resistance to apoptosis, and altered metabolic states^12,13^. Nevertheless, the association of SLC3A2 with gliomas and the potential biological effects in gliomas remain to be investigated.

This study delves into the expression of SLC3A2 in gliomas to assess its utility as a prognostic marker. Utilizing a robust bioinformatics approach, we analyze expression data from extensive cancer databases, striving to decipher the prognostic significance of SLC3A2 and its relationship with immune infiltration and metabolic pathways in glioma cells. Our investigation also correlates SLC3A2 expression with key clinical outcomes, such as patient survival and therapeutic response. Complementing our bioinformatics findings, we conducted validation using our clinical glioma samples collected independently, enhancing the study's translational relevance. Furthermore, to substantiate the role of SLC3A2 in glioma pathobiology, we performed both in vivo and in vitro experiments, providing a robust experimental framework to confirm our computational prognostic assessments and to explore the therapeutic potential of targeting SLC3A2 in gliomas.

In doing so, the research presented herein seeks to elucidate the multifaceted role of SLC3A2 in glioma pathobiology and its prognostic significance in gliomas through rigorous and accurate analyses.

## Materials and methods

### Downloading and processing relevant data

The relevant data for TCGA-LGG and TCGA-GBM were downloaded from The Cancer Genome Atlas (TCGA) (https://portal.gdc.cancer.gov) and compiled into TCGA-GBMLGG. The expression profile data of GSE4290, GSE50161, GSE43378, and GSE83300 were retrieved from the GEO database (https://www.ncbi.nlm.nih.gov/geo/). We obtained RNA-seq data of TCGA-GTEx-GBMLGG (n = 1846) processed uniformly through the Toil process from the UCSC XENA database. The processed dataset was utilized for group comparison and pan-cancer analysis. Chinese Glioma Genome Atlas (CGGA) database (http://www.cgga.org.cn) is an online tool that collects data related to more than 2,000 glioma samples in China. This study performed prognostic analysis on selected glioma patients (mRNAseq_325 dataset).

### Correlation analysis of differentially expressed genes

Based on the mid-level expression of SLC3A2, TCGA-GBMLGG samples were classified into SLC3A2^High^ and SLC3A2^Low^, The differential gene expression of the two groups was analyzed with the R packages DESeq2 with a cut-off of (*P* < 0.05, and |log2Fold Change (FC)|> 1)^14^.

### Functional enrichment analysis

Gene Ontology (GO) and Kyoto Encyclopedia of Genes and Genomes (KEGG) were utilized to conduct functional enrichment analyses on 596 differentially expressed genes (DEGs). The R package clusterprofiler was used to carry out GSEA enrichment analyses to determine if significant disparities exist in biological pathways between SLC3A2^High^ and SLC3A2^Low^ groups. Pathways were deemed statistically enriched if they had an adjusted P-value (P.adj) of less than 0.05 and a false discovery rate (FDR) of less than 0.25^15–17^.

### Protein–protein interaction (PPI) network composition of SLC3A2-associated

The STRING database (https://string-db.org/) has been utilized to excavate genes and proteins that are functionally intertwined with SLC3A2. The derived gene set data was implemented into Cytoscape for PPI network construction, MCC score calculation was accomplished using the cytoHubba plugin, and the top of SLC3A2 ten nodes with the highest MCC scores received as hub genes, which have been constructed into a hub gene network. Incorporation criterions have been defined as greater than 0.7 confidence threshold.

### DNA methylation analysis

To evaluate the prospective utility of SLC3A2 methylation-related loci in gliomas, we performed prognostic analyses by utilizing MethSurv (https://biit.cs.ut.ee/methsurv/), an accessible website for DNA methylation data.

### Analysis of immune infiltration and immune therapy response

The CIBERSORT method, supported by linear support vector regression, was utilized to calculate the compositions of 22 immunocyte cell types in each sample, ensuring that the proportion of all cell types summed up to equal one^18^. For evaluating immunotherapy responsiveness, the TIDE score, accessible through the TIDE database, was calculated for each sample, correlating higher scores with reduced survival and poor therapy outcomes^19,20^. Furthermore, EMT, a process pivotal for cellular transformation and metastasis, was studied in the context of the tumor microenvironment. We conducted ssGSEA using the "GSVA" package in R Studio to calculate EMT scores based on the "HALLMARK_EPITHELIAL_MESENCHYMAL_TRANSITION" gene set from the MSigDB database^21,22^. Additionally, the "GSVA" package enabled ssGSEA computation for 24 pre-established immune cell-related gene sets, aiming to determine individual cell type infiltration based on their respective enrichment scores^23^.

### Single-cell sequencing analysis

TISCH2 is an online database dedicated to investigating the tumor microenvironment in multiple cancers using single-cell sequencing (http://tisch.comp-genomics.org)^24^. In the study, the specific focus was on unraveling the tumor microenvironment of glioma through meticulous cell type annotation of three datasets (GSE102130, GSE70630, and GSE89567) utilizing the capabilities of TISCH2.

### Survival analysis

Survival analysis utilized the Kaplan–Meier method. To assess the influence of SLC3A2 and additional clinical factors on patient outcomes, Cox regression analyses were conducted^25^. Prognostic factors determined by univariate Cox regression analysis with *P* < 0.05 were subsequently analyzed in multivariate Cox regression to identify independent predictors of prognosis of gliomas.

### Meta-analysis

Published literature and relevant databases were searched for de-duplication of the included cohorts. HR with 95% CI was collected from the cohort and meta-analysis was performed. Meta-analysis was completed using STATA software.

### Prognostic significance evaluation

Nomograms incorporating independent prognostic features were created utilizing the R studio rms package^26^. The accuracy of the prediction of the nomograms was evaluated by calibration curves.

### Patients and sample

We collected tumor and surrounding tissue samples from 24 patients who had undergone curative surgery from 2017 to 2022 in the Second Hospital of Guangzhou Medical University. Research involving human participants was conducted following the ethical standards of the institution and the National Research Council. It adhered to the ethical standards of the 1964 Declaration of Helsinki and its subsequent amendments or similar. The Institutional Ethics Committee of the Second Hospital of Guangzhou Medical University approved this research (2020-YJS-KS-01). Informed consent was obtained from all of the individuals who participated in the study. For participants who were unable to obtain informed consent, the Research Ethics Committee granted exemptions due to the non-invasive nature of the study and the fact that the public interest of the study outweighed the need for individual consent.

### Cell lines

U87 and U251 cell lines were procured from iCell Bioscience Inc., located in Shanghai, China. The U87 and U251 cells were grown in Dulbecco's Modified Eagle Medium (DMEM) supplemented with 10% fetal bovine serum (FBS), both from Thermo Fisher Scientific, Inc., in a 37 ℃, 5% CO_2_ incubator.

### Transfection

Lentiviral shRNA constructs were sourced from GeneChem Co., Ltd., based in Shanghai, China. Following the manufacturer’s instructions, cells were transfected with the lentiviral particles. Cells were harvested 48 h post-transfection for subsequent analysis.

### Western blotting

Protein extracts were obtained by lysing cell lines with Pro-prep TM protein Extraction Solution (iNtRON Biotechnology, Korea). Equal protein quantities from each sample were subjected to electrophoresis using 10% SDS-PAGE gels for molecular weight-based separation. Following separation, the proteins were transferred onto PVDF membranes to prepare for detection. The membranes were blocked with 1 × TBST containing 5% BSA for 1 h at room temperature to minimize non-specific binding. The blots were then treated with primary antibodies (in a 5% BSA solution) at 4 °C overnight, and with secondary antibodies on the following day. The ECL chemiluminescence detection method (Pierce, Rockford, IL, USA) was employed to visualize protein bands of interest, facilitating the examination of target protein expression levels in the samples.

### Cell viability evaluation

A CCK-8 assay was utilized to evaluate cell viability. Cells under investigation were placed into 96-well plates, with each well containing 4,000 cells. After treatments, 10 μl of CCK-8 solution was added to each well, and the plates were incubated for 2 h at 37℃ in an environment of 95% air and 5% CO_2_. Absorbance at 450 nm was measured using a microplate reader. Results were expressed in terms of the percentage survival rate in comparison to the blank control.

### Assessment of colony formation

The clonogenic capability was assessed by performing colony formation assays on U87 and U251 cells. Post-seeding 500 cells per well in 6 cm dishes, the cells underwent a 24-h adherence phase at 37 °C in a 5% CO_2_ environment. Post various treatments, a 14-day incubation period ensued, with intermittent media refreshment every three days. Afterwards, colonies were fixed using a 3:1 methanol to acetic acid solution and stained with 0.5% crystal violet. A manual count was conducted for colonies, identified as cell clusters with no fewer than 50 cells, alternatively, a colony counter was utilized.

### Transwell assay

The migration and invasion capabilities of U87 and U251 cells were assessed using Transwell chambers that featured 8-µm pore-sized membranes (Corning, USA). In preparation, 20,000 cells in 200 µL of DMEM supplemented with 1% FBS were seeded into each well of the upper chamber, and 600 µL of DMEM containing 20% FBS was added to the lower chamber to establish a chemotactic gradient. For invasion assays, the upper membrane was precoated with 10 µL of Matrigel (BD Biosciences) to simulate the extracellular matrix barrier. After a 24-h incubation period, non-migratory or non-invasive cells were carefully removed from the membrane's upper surface. Cells that had migrated or invaded the underside of the membrane were fixed in ethanol and then stained with 0.2% crystal violet. Quantitative analysis was conducted by examining five randomly chosen fields under 100 × magnification. The experimental procedure was repeated three times to ensure the results' reliability and consistency.

### Xenograft tumor assay

Female 6-week-old BALB/c nude mice, sourced from the Guangdong Medical Laboratory Animal Center in Guangzhou, China, were intracranially injected with 1 × 10^6 U87 cells in 4 µL PBS (six mice per group). The injections were performed using a Micro 4 Microsyringe Pump Controller (World Precision Instruments, Sarasota, FL) connected to a Hamilton syringe equipped with a 33-gauge needle (Hamilton, Reno, NV), targeting the mid-right striatum at specified coordinates from bregma: + 0.5 anterior–posterior, + 2.0 medio-lateral, -2.8 dorso-ventral. Daily observations were made for any signs of death or neurological symptoms in the mice, which were then euthanized upon the appearance of such symptoms. Following euthanasia, brains were harvested, fixed in 4% PFA, encased in paraffin, and sliced coronally from the front to the back. Tumor size was determined by selecting the largest cross-sectional area of the tumor for measurement. Tumor volume was calculated using the formula V = (a × b^2) / 2, where 'a' represents the longest diameter and 'b' the shortest, with 'a' and 'b' being measured via Image J. Survival analysis involved the intracranial injection of mice (six per group) following the procedure outlined above. Mice nearing death were euthanized under deep anesthesia, and the survivors were euthanized 30 days post-injection of U87 cells. The Institutional Animal Care and Use Committee at Guangzhou Medical University granted approval for all experiments involving mice (No.A2020-B18). All methods were performed in accordance with the ARRIVE guidelines and related guidelines and regulations.

### Statistical analysis

R studio (version 4.2.2) and SPSS (Statistics 25) were employed for statistical analysis, with in vitro study data showing mean ± standard error from three separate experiments. T-test or Wilcoxon rank-sum test was conducted for inter-group comparisons, while one-way ANOVA or Kruskal–Wallis test was conducted for multi-group comparisons. *P* < 0.05 represented statistical significance.

## Results

### Correlation analysis of disulfidptosis-related genes in gliomas

**Figure 1:**
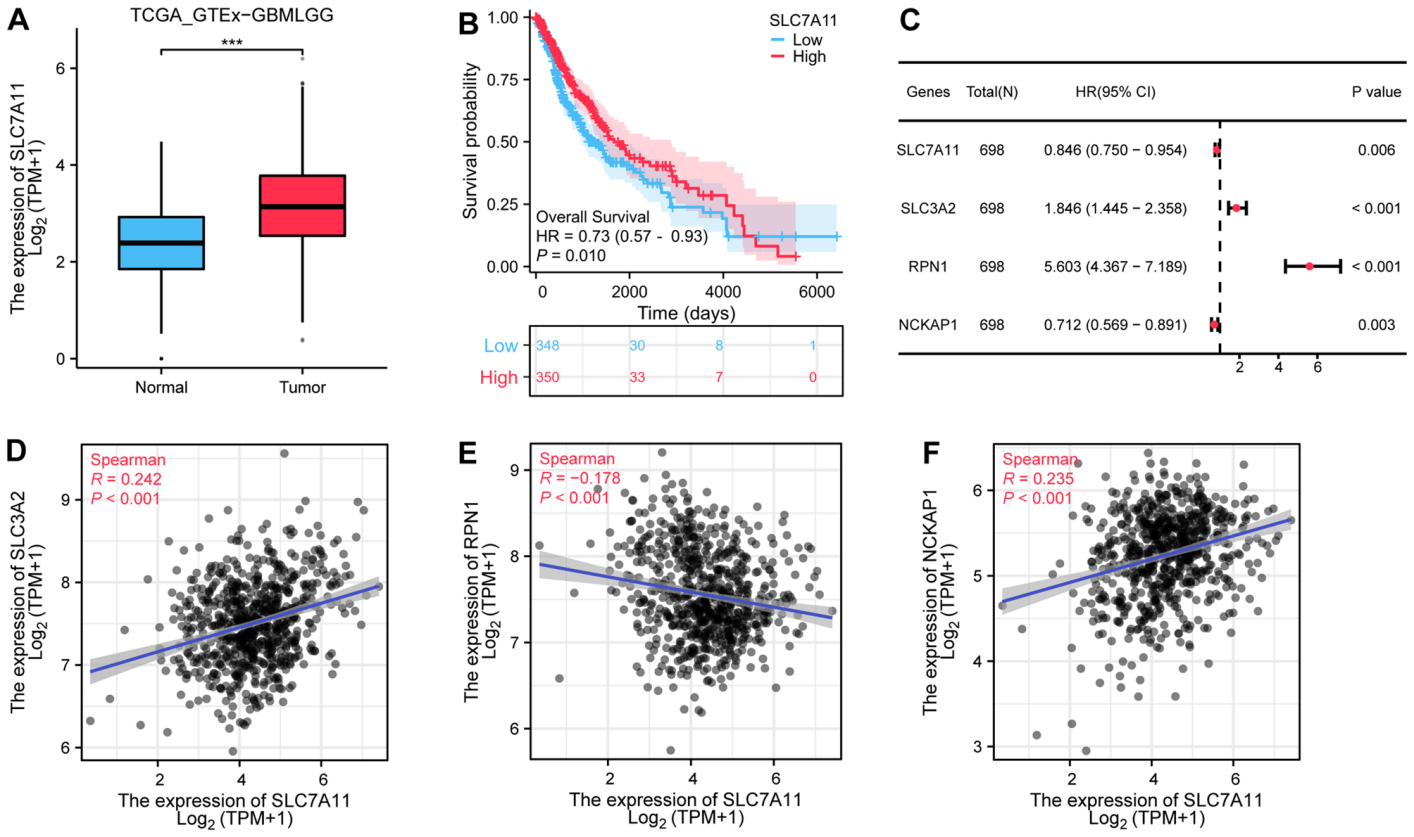
Expression of disulfidptosis-related genes in TCGA-GBMLGG. (**A**) The expression level of SLC7A11, a critical gene of disulfidptosis, in the TCGA dataset. (**B**) High SLC7A11 expression corresponds to a favorable prognosis for gliomas. (**C**) SLC3A2, RPN1, and NCKAP1 are associated with prognosis. (**D**–**F**) The correlation between SLC3A2, RPN1, NCKAP1, and SLC7A11. *** *P* < 0.001.

Figure 1A-B illustrated a pronounced upregulation of SLC7A11 in tumor samples within the TCGA-GTEx-GBMLGG dataset (*P* < 0.001), with a consequential divergence in survival probability between cohorts characterized by high versus low SLC7A11 expression (*P* = 0.010). Univariate Cox regression analysis has discerned significant associations between glioma patient survival trajectories and the expression profiles of several disulfidptosis-related genes, specifically SLC3A2, RPN1, NCKAP1, and SLC7A11 (Fig. 1C, all, *P* < 0.05). Spearman correlation analyses were employed to elucidate the interplay between SLC3A2, RPN1, NCKAP1, and SLC7A11 within the glioma landscape (Fig. 1D-F), revealing the strongest correlation between SLC3A2 and SLC7A11 (R = 0.242, *P* < 0.001). Predicated on these compelling insights, SLC3A2 was earmarked for more exhaustive investigative scrutiny.

### Assessing expression levels of SLC3A2 in gliomas

**Figure 2:**
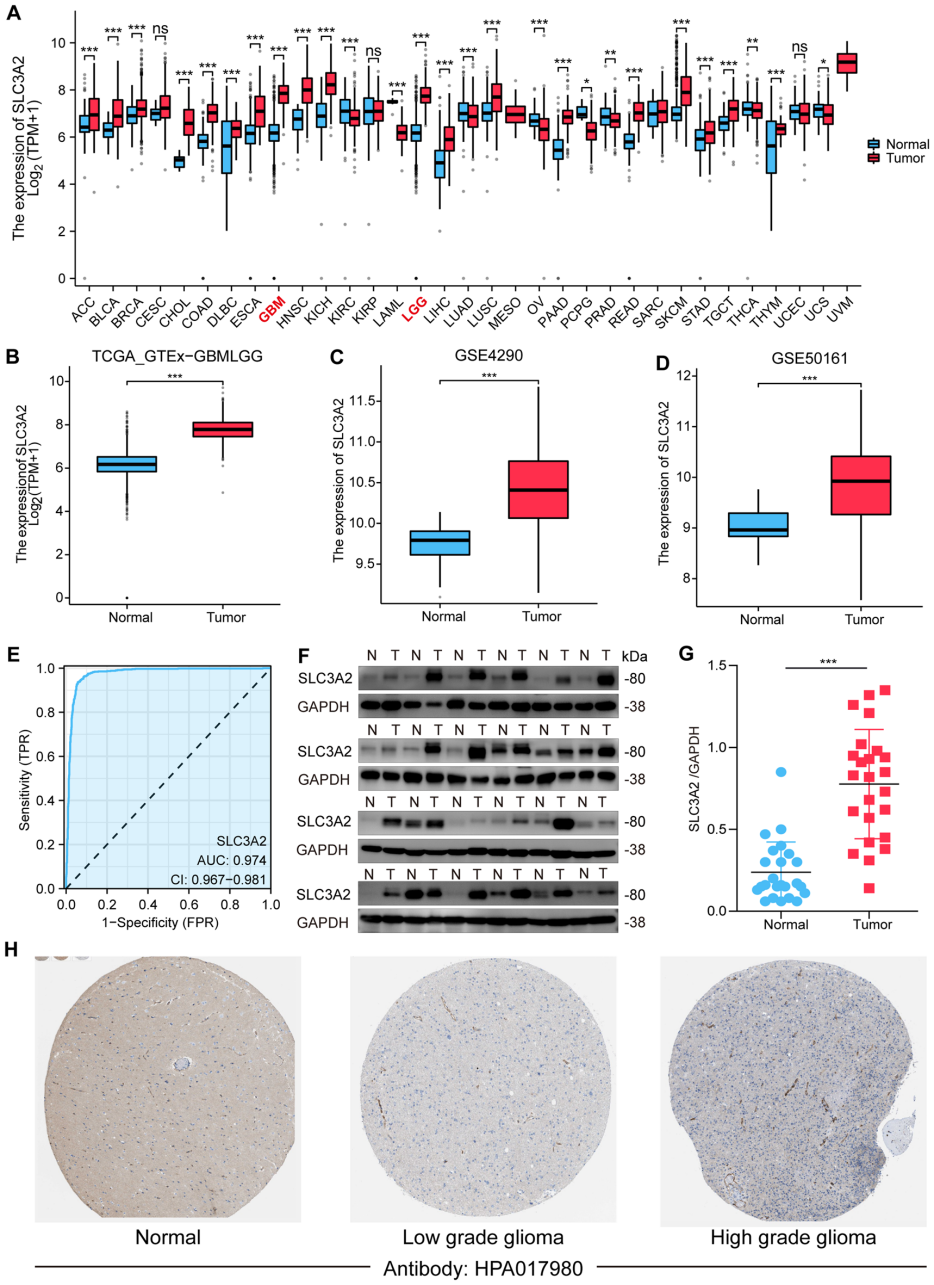
Expression levels of SLC3A2 in various categories of tumors and gliomas. (**A**) Comparison of SLC3A2 expression between different categories types and normal samples. (**B**–**D**) Expression levels of SLC3A2 in TCGA-GTEx-GBMLGG, GSE4290, and GSE50161 in tumor samples and normal samples. (**E**) A diagnostic ROC curve of SLC3A2 in gliomas. (**F**–**G**) The western blot results of SLC3A2 expression in 24 patients with glioma. (**H**) Immunohistochemical results of SLC3A2 in the HPA database. * *P* < 0.05, ** *P* < 0.01, *** *P* < 0.001, ns not significant.

An analysis across various cancer types highlighted a consistent increase in SLC3A2 mRNA levels, including in READ, TGCT, and HNSC (Fig. 2A). Notably, pronounced disparities were also discernible in low-grade gliomas (LGG) and glioblastoma (GBM). To deepen our understanding of SLC3A2 mRNA levels in GBMLGG, we scrutinized the TCGA-GTEx-GBMLGG, GSE4290, and GSE50161 datasets. These analyses illuminated a marked ascendancy in SLC3A2 mRNA within tumor specimens relative to normal tissues (Fig. 2B-D, P < 0.001 for all datasets). Moreover, diagnostic ROC analysis showcased the superior predictive accuracy of SLC3A2 in gliomas, evidenced by an Area Under the ROC Curve (AUC) of 0.974 (95% CI: 0.967–0.981), affirming its competency in differentiating glioma tissues from normal counterparts (Fig. 2E). Consistent with the TCGA and GEO database findings, an upregulation of SLC3A2 was observed in glioma tissues in our collection of 24 paired tumor and adjacent non-tumor tissues from glioma patients (Fig. 2F-G). Immunohistochemical analysis has indicated high levels of SLC3A2 expression in tumor tissues, and the highest levels of SLC3A2 expression have been observed in high-grade gliomas (Fig. 2H).

### Association of SLC3A2 with clinicopathological features in gliomas

Table 1 revealed significant deviations in the composition ratios of six clinicopathological parameters, including WHO grade (G2&G3 vs. G4), IDH status, Histological type, Age, OS event, and DSS event (all, *P* < 0.001) among cohorts exhibiting high versus low SLC3A2 expression. Logistic regression analysis outcomes (Table 2) also highlighted notable clinicopathological distinctions between high and low SLC3A2 expressers regarding WHO grade (G4 vs. G2&G3, OR = 2.184, 95%CI [1.522–3.157], *P* < 0.001), 1p/19q codeletion (non-codel vs. codel, OR = 0.674, 95%CI [0.474–0.953], *P* = 0.026), IDH status (Mut vs. WT, OR = 0.349, 95%CI [0.251–0.483], *P* < 0.001), and age (> 60 vs. <  = 60, OR = 1.503, 95%CI [1.039–2.187], *P* = 0.032).

**Table 1:** Clinicopathological profiles of SLC3A2^High^ and SLC3A2^Low^.

Characteristics	Low expression of SLC3A2	High expression of SLC3A2	*P* value
n	349	349	
WHO grade, n (%)			** < 0.001**
G2&G3	243 (35%)	168 (24.2%)	
G4	104 (15%)	180 (25.9%)	
IDH status, n (%)			** < 0.001**
Mutant	262 (38.1%)	180 (26.2%)	
WT	81 (11.8%)	165 (24%)	
1p/19q codeletion, n (%)			0.082
non-codel	271 (39.2%)	249 (36%)	
codel	76 (11%)	95 (13.7%)	
Primary therapy outcome, n (%)			0.057
CR	85 (18.3%)	54 (11.6%)	
PR	33 (7.1%)	32 (6.9%)	
PD	51 (11%)	61 (13.1%)	
SD	87 (18.8%)	61 (13.1%)	
Histological type, n (%)			** < 0.001**
Astroctyoma	176 (25.6%)	95 (13.8%)	
Glioblastoma	42 (6.1%)	204 (29.7%)	
Oligodendroglioma	130 (18.9%)	41 (6%)	
Gender, n (%)			0.193
Male	192 (27.5%)	209 (29.9%)	
Female	157 (22.5%)	140 (20.1%)	
Age, n (%)			** < 0.001**
< = 60	320 (45.8%)	235 (33.7%)	
> 60	29 (4.2%)	114 (16.3%)	
OS event, n (%)			** < 0.001**
Alive	349 (50%)	77 (11%)	
Dead	0 (0%)	272 (39%)	
DSS event, n (%)			** < 0.001**
Alive	349 (51.6%)	84 (12.4%)	
Dead	0 (0%)	244 (36%)	

Bold values indicate *P* value < 0.05

**Table 2:** Logistic analysis of the correlation between clinicopathologic profiles and SLC3A2 expression level in GBMLGG patients.

Characteristics	Total (N)	Odds Ratio (OR)	*P* value
WHO grade (G4 vs. G2&G3)	635	2.184 (1.522–3.157)	** < 0.001**
1p/19q codeletion (non-codel vs. codel)	689	0.674 (0.474–0.953)	**0.026**
IDH status (Mut vs. WT)	686	0.349 (0.251–0.483)	** < 0.001**
Primary therapy outcome (PR&CR vs. PD&SD)	462	0.962 (0.664–1.393)	0.839
Gender (Female vs. Male)	696	0.932 (0.690–1.258)	0.646
Race (Black or African American&White vs. Asian)	683	0.610 (0.183–1.848)	0.391
Age (> 60 vs. < = 60)	696	1.503 (1.039–2.187)	**0.032**

Bold values indicate *P* value < 0.05

### Pathway enrichment analysis of DEGs based on SLC3A2 in gliomas

**Figure 3:**
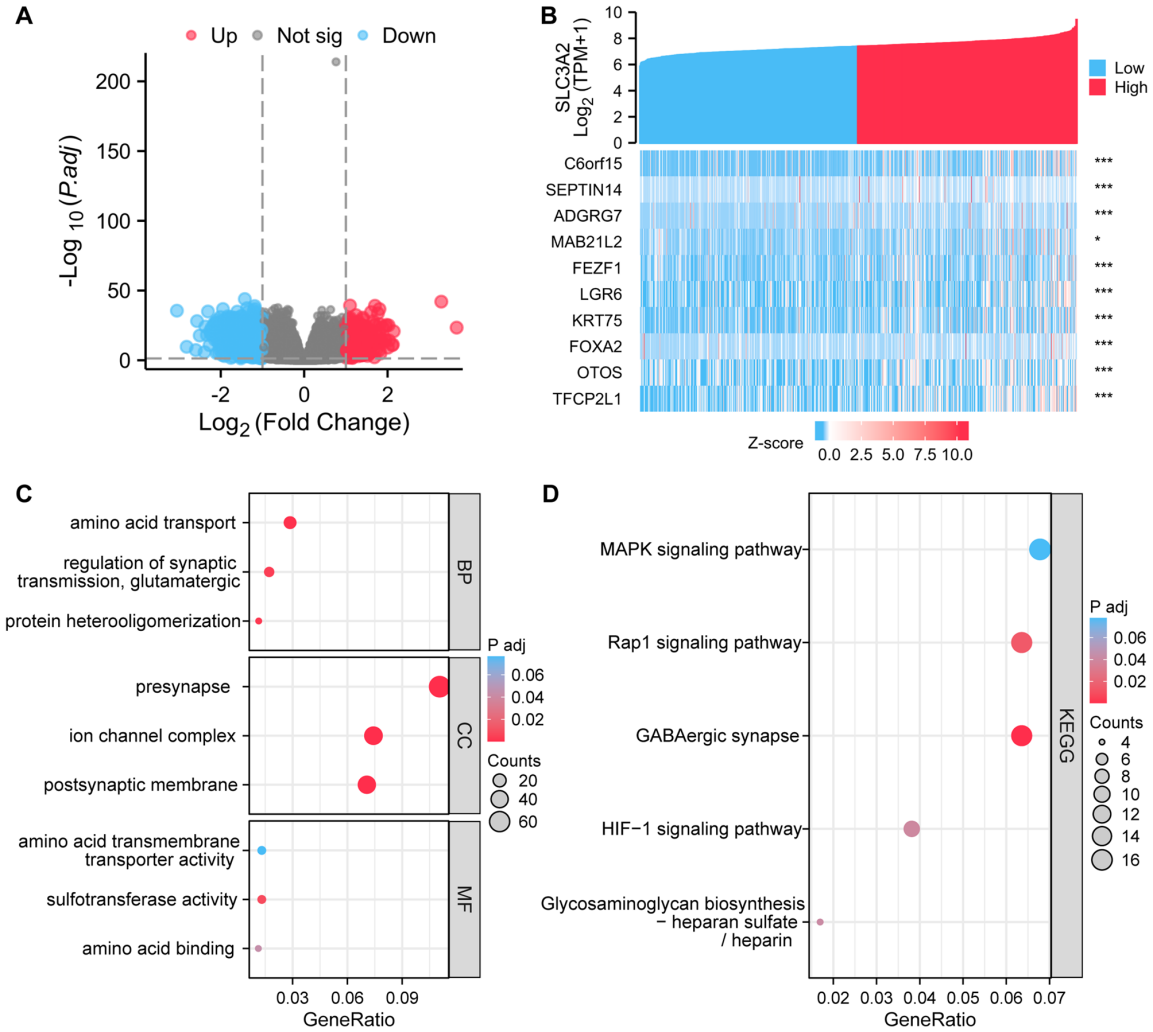
Pathway enrichment analysis of the DEGs based on the SLC3A2 in gliomas. (**A**) Volcano plot showing the distribution of DEGs, with red dots representing significantly upregulated DEGs and blue dots representing significantly downregulated DEGs. (**B**) Co-expression heatmap between SLC3A2 expression and the top 10 DEGs. (**C**–**D**) Bubble plots showing the results of GO and KEGG pathway analysis of DEGs.

In TCGA datasets of glioma, a grand total of 596 DEGs associated with SLC3A2 were detected. Out of these, 144 DEGs (24%) were upregulated and 452 DEGs (76%) were downregulated (cut off: |log2FC|> 1, P.adj < 0.05) (Fig. 3A). Subsequently, the top ten DEGs, namely C6orf15, SEPTIN14, ADGRG7, MAB21L2, FEZF1, LGR6, KRT75, FOXA2, OTOS, and TFCP2L1, were selected to construct a co-expression heatmap, as depicted in Fig. 3B.

**Figure 4:**
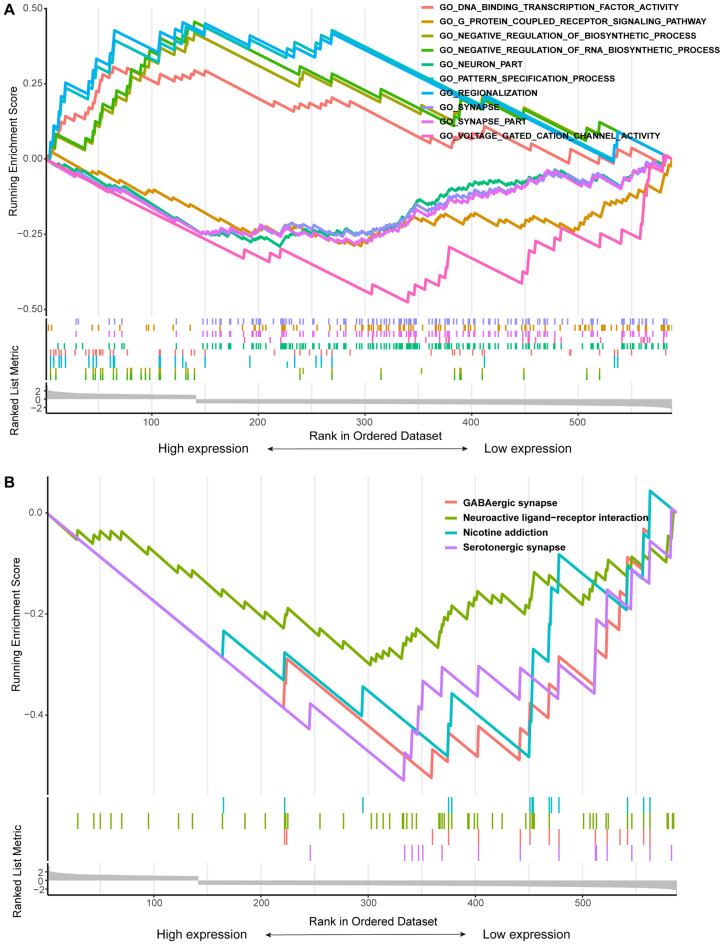
Pathway enrichment analysis of the DEGs based on the SLC3A2 in gliomas. (**A**–**B**) GSEA outcomes for SLC3A2^High^ and SLC3A2^Low^ gliomas. The results of the GSEA analysis were derived from the FDR, NES, and adjusted P-value metrics.

To gain further insight into the potential functional pathways involving the 596 DEGs, comprehensive GO, KEGG, and GSEA analyses were performed. The GO enrichment analysis, encompassing biological processes (BP), cellular components (CC), and molecular functions (MF), unveiled significant associations of the DEGs with diverse GO pathways. Notably, enrichment was observed in BP, including amino acid transport, regulation of synaptic transmission, glutamatergic, and protein heterooligomerization. In terms of CC, enrichment was detected in presynapse, ion channel complex, and postsynaptic membrane. Moreover, MF analysis revealed significant enrichment in sulfotransferase activity, amino acid transmembrane transporter activity, and amino acid binding. Among them amino acid transport, sulfotransferase activity, and amino acid binding are intricately linked to sulfur and amino acid metabolism, suggesting their potential involvement in pathways associated with disulfidptosis (Fig. 3C). Importantly, this study elucidated that SLC3A2 expression may regulate glioma development through the process of disulfidptosis. KEGG enrichment analysis highlighted distinct pathways enriched in DEGs, including the MAPK signaling pathway, Rap1 signaling pathway, GABAergic synapse, HIF-1 signaling pathway, and Glycosaminoglycan biosynthesis—heparan sulfate / heparin (Fig. 3D). Furthermore, GSEA was employed to investigate the enrichment of GO and KEGG pathways in SLC3A2^High^ and SLC3A2^Low^ glioma (P. adjust < 0.05, FDR < 0.25). In SLC3A2^High^ glioma, the top five significantly enriched GO pathways included Negative regulation of RNA biosynthetic process, Negative regulation of biosynthetic process, Pattern specification process, Regionalization, and DNA binding transcription factory activity. Conversely, in SLC3A2^Low^ glioma, the top five enriched GO pathways comprised Neuron part, Voltage gated cation channel activity, Synapse part, G-protein coupled receptor signaling pathway, and Synapse. Regarding KEGG pathway enrichment, the low expression group of SLC3A2 exhibited significant enrichment in Serotonergic synapse, GABAergic synapse, Neuroactive ligand-receptor interaction, and Nicotine addiction (Fig. 4A-B, Table S1). These pathways possess a close association with tumor cell proliferation and migration. Thus, SLC3A2 holds the potential to influence tumor cell proliferation, migration, and metabolism.

### Protein–protein interaction network composition of SLC3A2 and its associated genes in gliomas

**Figure 5:**
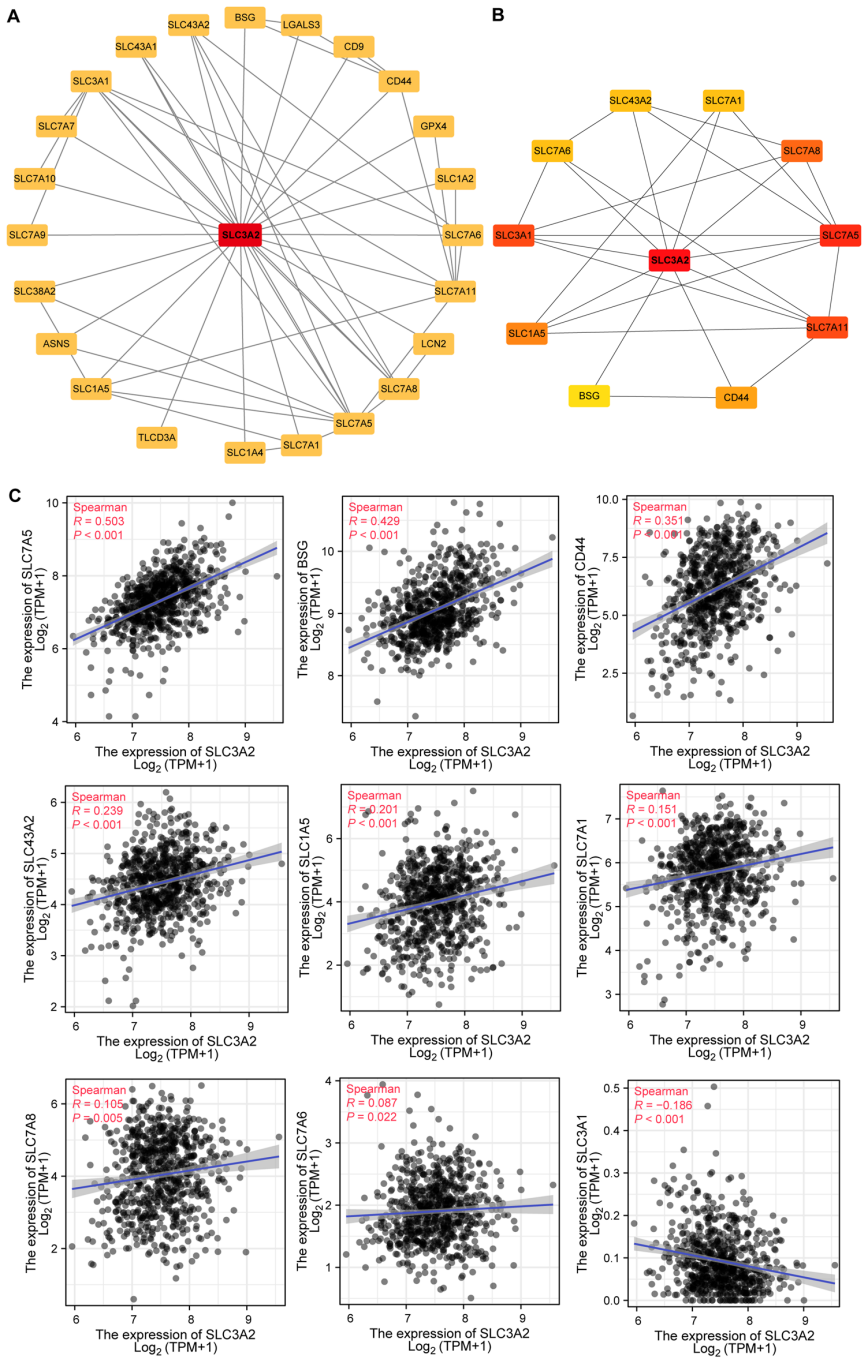
PPI network and hub genes determination. (**A**) PPI network constructed by SLC3A2-related genes. (**B**) 10 hub genes associated with SLC3A2. (**C**) Correlation analyses of SLC3A2 and hub genes.

The SLC3A2-related PPI network in gliomas has been constructed using Cytoscape (Fig. 5A). The related hub genes were filtered out in accordance with the calculation results of MCC (Fig. 5B). The significant inter-correlations between the hub genes of SLC3A2 and SLC3A2 have been observed in all of them (Fig. 1D, Fig. 5C). Remarkably, SLC7A11 was also one of the related hub genes of SLC3A2, which reinforced the conclusion that SLC3A2 may interact with gliomas through disulfidptosis.

### The impact of SLC3A2 DNA methylation on glioma prognostication

**Figure 6:**
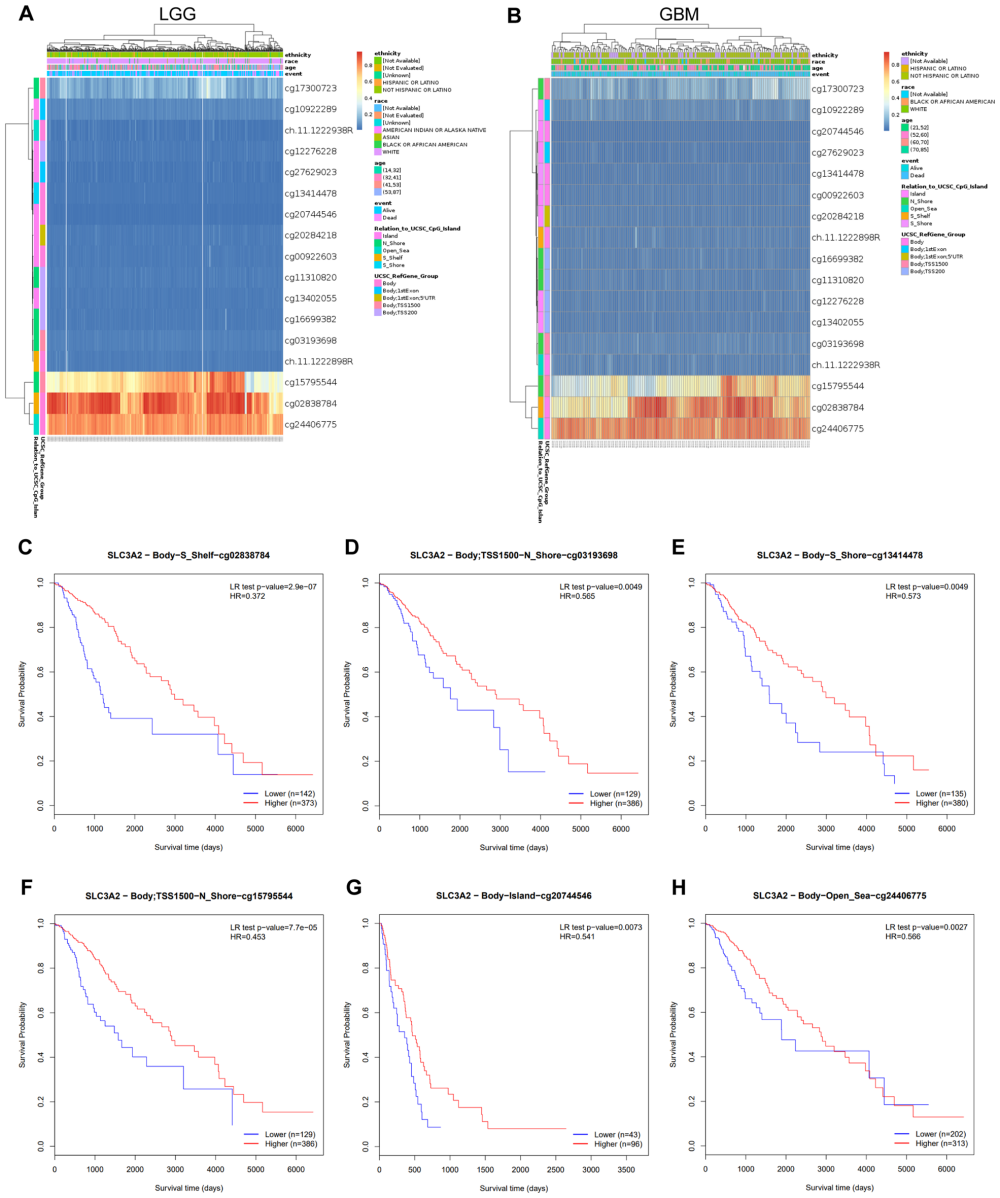
DNA methylation levels of SLC3A2 in gliomas and their association with the survival prognosis of glioma patients. (**A**–**B**) Waterfall plots highlight the relationship between the methylation level of the SLC3A2 promoter and the gene expression level. (**C**–**H**) Prognostic analyses of methylation loci correlating with SLC3A2.

DNA methylation represents a fundamental epigenetic mechanism that orchestrates gene expression and influences oncogenesis. Our investigation into the interplay between SLC3A2 methylation and its transcriptional activity within the GBMLGG cohort unveiled that heightened methylation coincides with diminished gene expression (Fig. 6A-B). Moreover, we pinpointed several CpG loci (cg02838784, cg03193698, cg13414478, cg15795544, cg20744546, and cg24406775) within the SLC3A2 promoter domain that act as prognostic sentinels (all, HR < 1, *P* < 0.05), wherein their hypermethylation is concomitant with favorable clinical outcomes (Fig. 6C-H).

### Interrelation of SLC3A2 expression and immune infiltration levels

**Figure 7:**
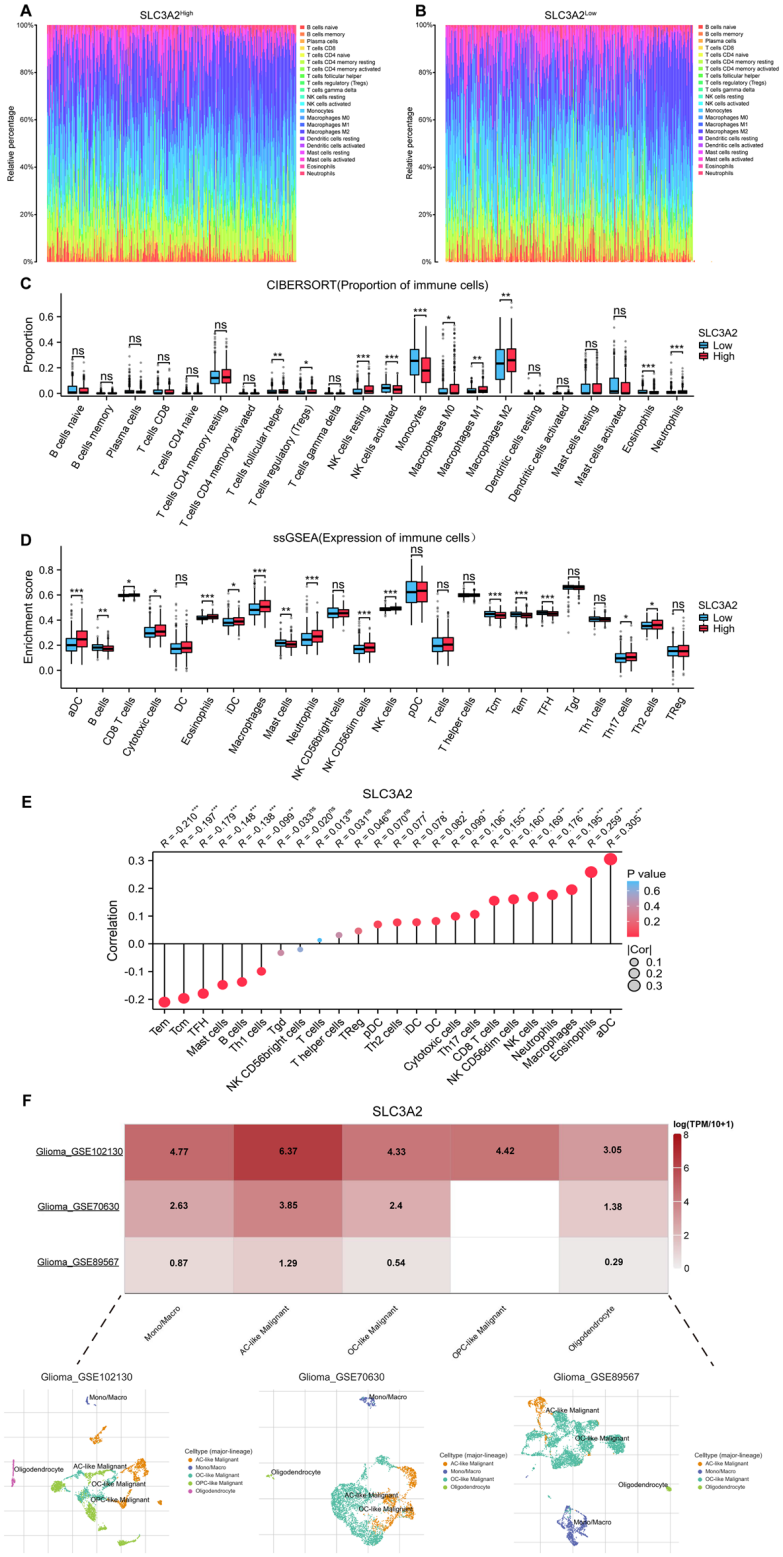
Immune cell infiltration analysis in gliomas associated with SLC3A2 expression. (**A**–**B**) Composite bar diagrams display the immune cell proportions in the SLC3A2^High^ and SLC3A2^Low^. (**C**) Based on CIBERSORT results, the box plot shows the comparison of 22 immune cell types between SLC3A2^High^ and SLC3A2^Low^. (**D**) A box plot based on ssGSEA results shows the comparison of enrichment scores for 24 immune cells categories between SLC3A2^High^ and SLC3A2^Low^. (**E**) Association between infiltration levels of 24 immune cells and SLC3A2 expression. (**F**) Summary of three single-cell sequencing datasets correlate with SLC3A2 expression, with UMAP plots representing individual cells colored by cell clusters. * *P* < 0.05, ** *P* < 0.01, *** *P* < 0.001, ns not significant.

Utilizing the LM22 gene signature and CIBERSORT analytical tool, we differentiated the abundance of 22 leukocyte subsets between samples expressing divergent levels of SLC3A2, as depicted in the composite bar diagram (Fig. 7A-B). Notably, a higher incidence of T cells (follicular helper and regulatory), NK cells resting, M0/M1/M2 macrophages, and Neutrophils was identified in SLC3A2^High^ samples, contrasted by an escalated presence of activated NK cells activated, Monocytes, and Eosinophils in SLC3A2^Low^ counterparts (Fig. 7C). Subsequent ssGSEA evaluation affirmed substantial variances in the enrichment scores of numerous immune subsets between the two groups (Fig. 7D). Simultaneously with this inquiry, we conducted the ssGSEA assay to decipher the connection between SLC3A2 expression and leukocyte infiltration dynamics, encapsulated visually through a lollipop plot crafted with the ggplot2 package (Fig. 7E). A significant positive association emerged with aDC (R = 0.305, *P* < 0.001), Eosinophils (R = 0.259, *P* < 0.001), Macrophages (R = 0.195, *P* < 0.001), and others, contrasted with a negative affinity towards Tem (R = − 0.210, *P* < 0.001), Tcm (R = − 0.197, *P* < 0.001), TFH (R = − 0.179, *P* < 0.001), among others.

**Figure 8:**
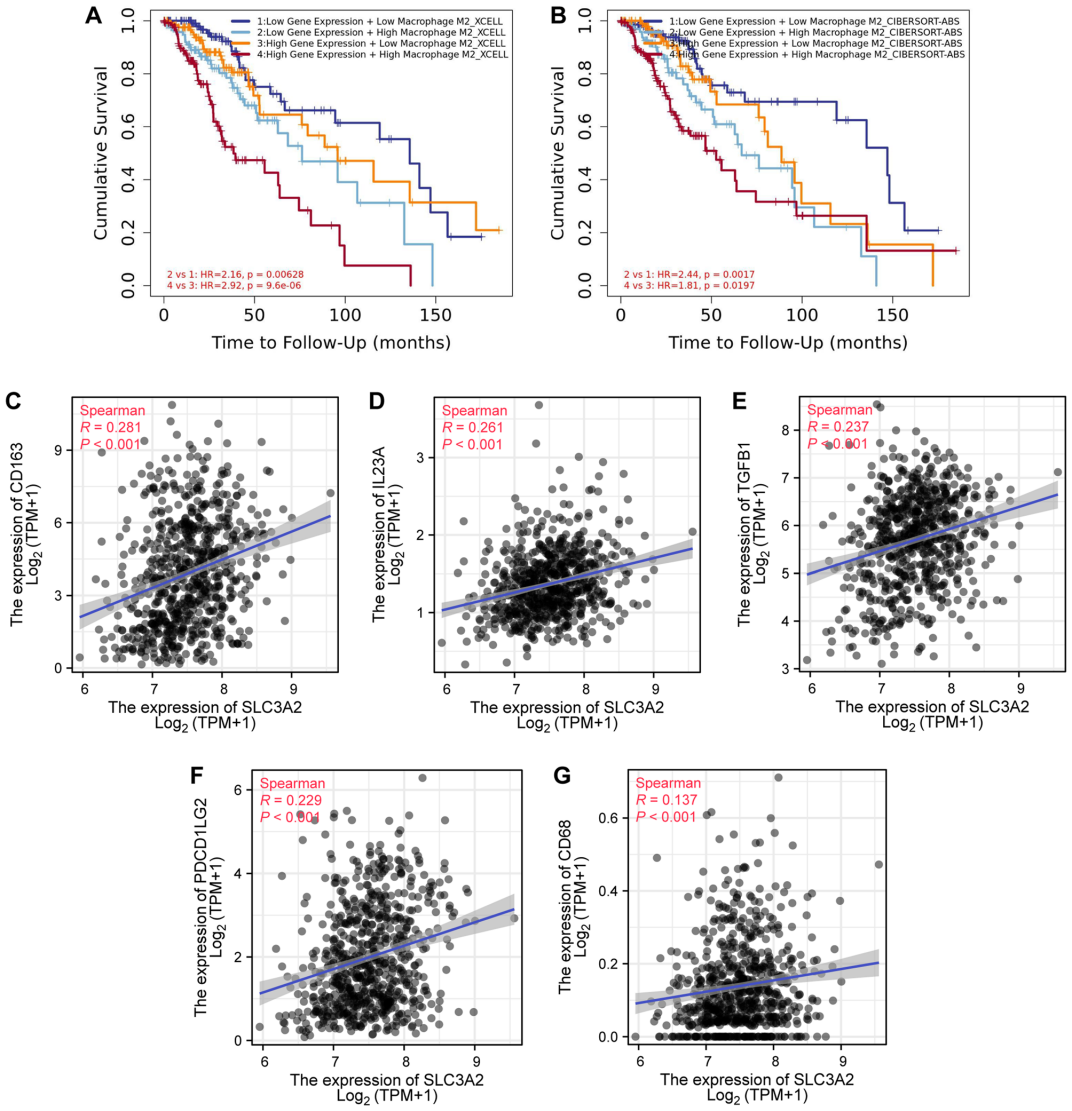
Correlation between SLC3A2 expression levels and M2 macrophage markers. (**A**–**B**) Poor prognosis of gliomas is associated with increased M2 macrophage infiltration. (**C**–**G**) SLC3A2 is significantly positively correlated with five common M2 macrophage markers.

Expanding our analytical horizon, single-cell sequencing scrutiny of datasets GSE102130, GSE70630, and GSE89567 divulged a predominant expression of SLC3A2 in Monocytes/Macrophages, AC-like Malignant, and OC-like Malignant cells (Fig. 7F, Figure S1). Subsequent corroboration via the Timer 2.0 platform affirmed the negative prognostic interplay between SLC3A2^High^ and M2 macrophage infiltration (Fig. 8A-B). This led to an examination of the concordance between SLC3A2 and quintessential M2 macrophage markers, namely CD163, IL23A, TGFB1, PDCD1LG2, and CD68, unveiling a significant co-expression landscape (Fig. 8C-G).

Synthesizing the insights from immune infiltration metrics, single-cell transcriptomics, and M2 marker co-expression analyses, we postulate that SLC3A2 catalyzes a transformative effect on the glioma tumor microenvironment, particularly by steering macrophage polarization towards an M2 phenotype.

### Association between SLC3A2 expression and indicators related to tumor immune response

**Figure 9:**
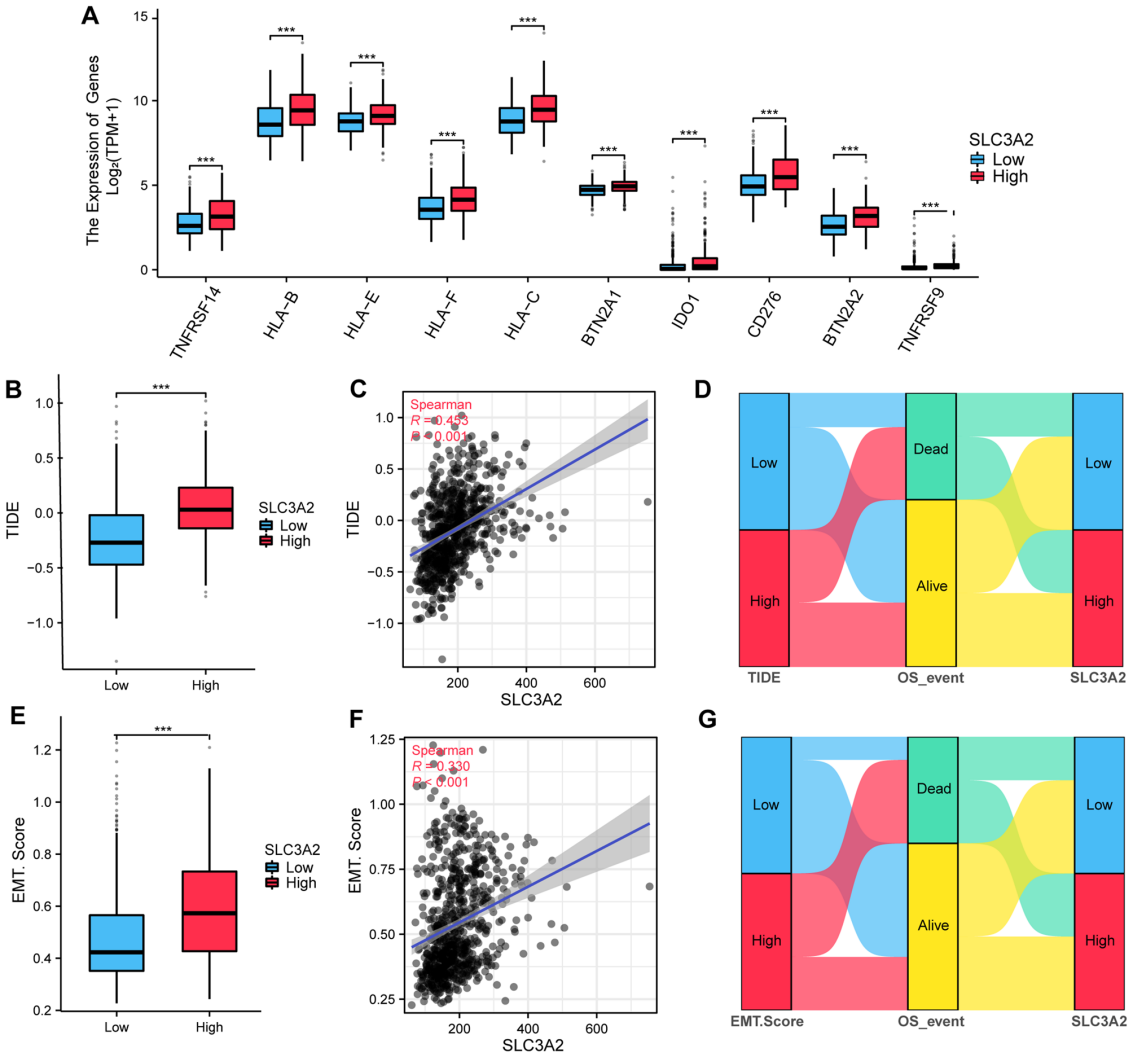
Association between SLC3A2 expression and indicators related to tumor immune response. (**A**) Expression levels of common immune checkpoints (ICPs) in SLC3A2^High^ and SLC3A2^Low^. (**B**–**C**) TIDE score is significantly positively correlated with SLC3A2 expression levels. (**D**) Sankey diagram showing the association between TIDE, OS events, and SLC3A2 expression levels. (**E**–**F**) Significant positive correlation between EMT-Score and SLC3A2 expression levels. (**G**) Sankey diagram illustrates the relationship between EMT-scores, OS events, and SLC3A2 expression levels. * *P* < 0.05, ** *P* < 0.01, *** *P* < 0.001.

In scrutinizing the differential expression profiles of pivotal immune checkpoint proteins (ICPs) across SLC3A2 expression spectra, we discerned pronounced upregulation of several ICPs including TNFRSF14, HLA-B, HLA-C, BTN2A1, IDO1, CD276, HLA-F, BTN2A2, HLA-E and TNFRSF9 in SLC3A2^High^ specimens (all, *P* < 0.001) (Fig. 9A). Spearman's correlation coefficients, calculated within the TCGA-GBMLGG framework, unveiled a consistent positive affiliation between SLC3A2 and the aforementioned ICPs (Figure S2), suggesting patients with augmented SLC3A2 expression potentially harbor enhanced autoimmune self-tolerance and fortified peripheral immune modulatory competencies. A profound exploration of immune responsiveness revealed an amplified Tumor Immune Dysfunction and Exclusion (TIDE) parameter in SLC3A2^High^ patients, suggesting suboptimal immunotherapeutic efficacy and heightened tumor immune evasion (Fig. 9B, *P* < 0.001). This inference is bolstered by a robust Spearman correlation underscoring the parallelism between increasing SLC3A2 expression and TIDE scores, implying enhanced tumor escape mechanisms and deteriorating prognoses (Fig. 9C, R = 0.453, *P* < 0.001). Concordantly, a higher mortality index among patients with elevated TIDE scores corroborates these findings (Fig. 9D). Focusing on the epithelial-mesenchymal transition (EMT), a pivotal facilitator of cellular metastatic and immunosuppression and immune evasion dissemination, we discerned a notable discrepancy in EMT scores between high and low SLC3A2 expressers (Fig. 9E, *P* < 0.001). A significant positive trajectory was established between SLC3A2 expression and EMT scores (Fig. 9F, R = 0.330, *P* < 0.001), substantiated further by Sankey diagram analysis indicating a heightened mortality predisposition in patients with escalated EMT scores, thereby underscoring the prognostic gravity of EMT-facilitated oncogenic progression (Fig. 9G).

### Prognostic significance of SLC3A2 expression in gliomas

**Figure 10:**
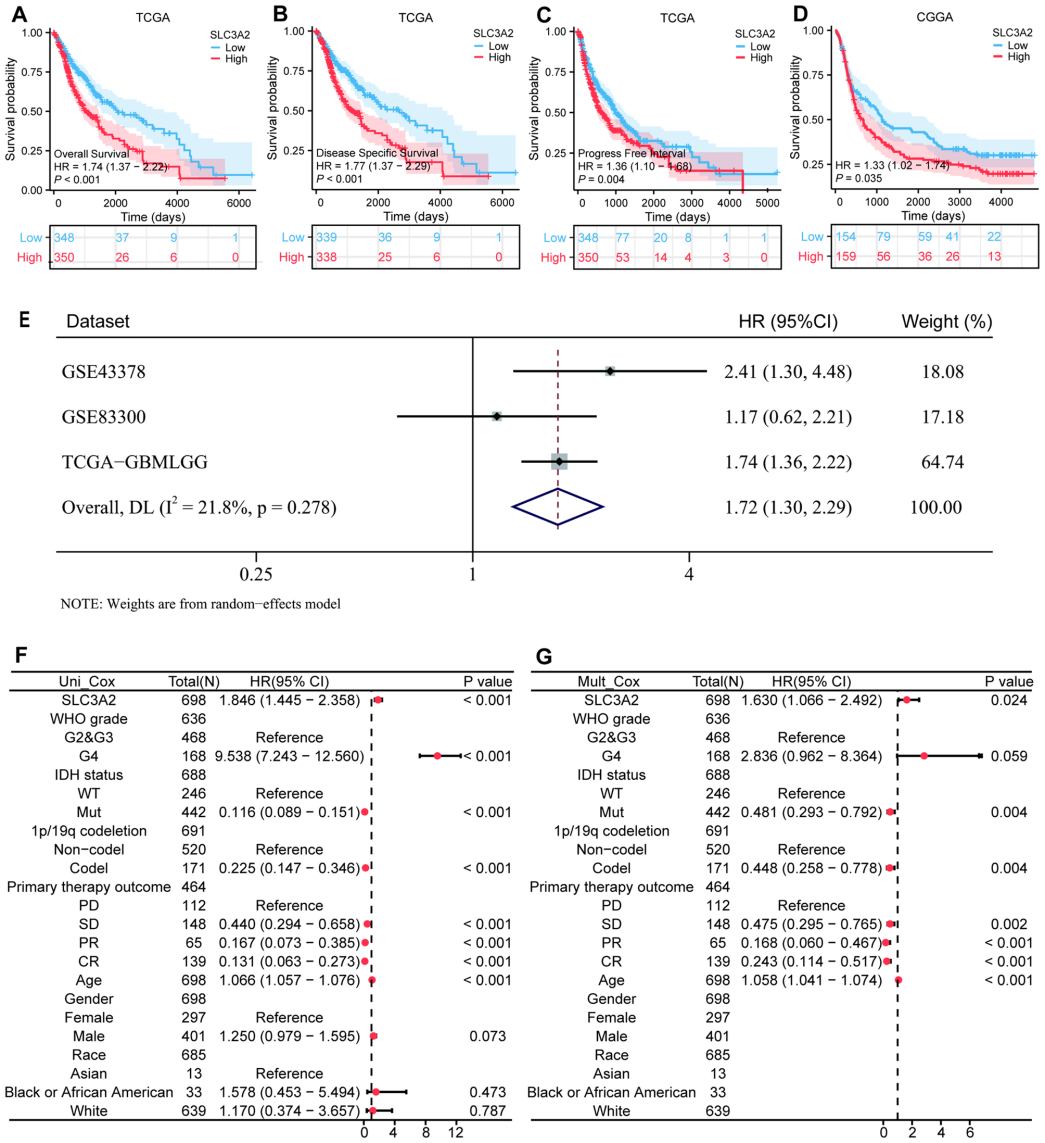
Assessment of the prognostic implications of SLC3A2 in gliomas. KM plots show the difference in (**A**) OS, (**B**) DSS, and (**C**) PFI between SLC3A2^High^ and SLC3A2^Low^ glioma samples in the TCGA-GBMLGG. (**D**) KM plot demonstrates the difference in OS between SLC3A2 ^High^ and SLC3A2 ^Low^ glioma samples in the CGGA dataset. (**E**) Forest plot of highly expressed SLC3A2 with OS for three cohorts. (**F**–**G**) OS forest maps based on Univariate Cox regression analysis and Multivariate Cox regression analysis. HR, hazard ratio; CI, confidence interval.

To elucidate the prognostic impact of SLC3A2 on glioma, we employed the survival analysis to interrogate data from TCGA-GBMLGG and CGGA patient cohorts. Distinctly, patients within the SLC3A2^High^ category manifested significantly deteriorated overall survival (OS), disease-specific survival (DSS), and progress free interval (PFI) corroborated by a hazard ratio (HR) of 1.74 (95% CI, 1.37–2.22, *P* < 0.001), 1.77 (95% CI, 1.37–2.29, *P* < 0.001), and 1.36 (95%CI, 1.10–1.68, *P* < *P* < 0.01) respectively in TCGA-GBMLGG, and compromised OS (HR = 1.33, 95% CI [1.02–1.74], *P* < 0.05) in the CGGA dataset (Fig. 10A-D). A meta-analysis spanning the three cohorts reinforced these findings, indicating a unified HR of 1.72 (95% CI, 1.30–2.29) and negligible heterogeneity (I^2^ = 21.8%), thus firmly establishing high SLC3A2 expression as a harbinger of poor glioma prognosis (Fig. 10E).

Furthering our understanding, univariate and multivariate Cox regression analyses delineated SLC3A2 alongside other clinicopathological indicators as salient prognostic constituents within TCGA-GBMLGG (Fig. 10F-G, Table S2). Specifically, multivariate analysis pinpointed SLC3A2 (HR = 1.630, 95% CI [1.066–2.492], *P* = 0.024), IDH status (HR = 0.481, 95% CI [0.293–0.792], *P* = 0.004), 1p/19q codeletion (HR = 0.448, 95% CI [0.258–0.778], *P* = 0.004), Primary therapy outcome, and Age (HR = 1.058, 95% CI [1.041–1.074], *P* < 0.001) as independent prognostic arbiters for glioma OS.

**Figure 11:**
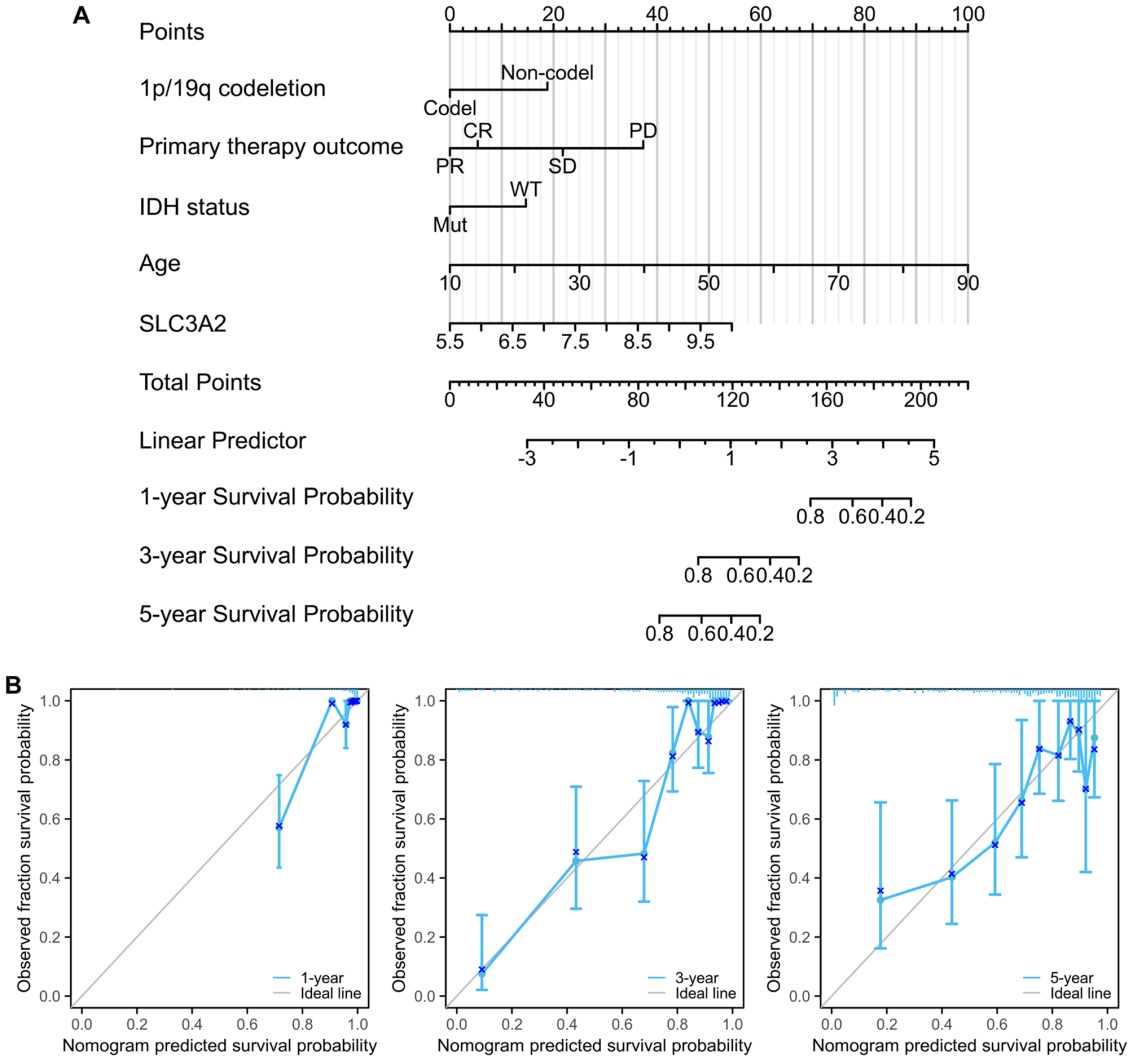
Construction and validation of the nomogram. (**A**) A nomogram for predicting overall survival rates. (**B**) Calibration curves for survival prediction nomogram for glioma patients.

Aiming for enhanced precision in prognostic forecasts, we devised a nomogram integrating the aforementioned independent prognostic determinants for OS. The cumulative point system—incorporating age, SLC3A2 expression, 1p/19q codeletion, IDH status, and primary therapy outcome—directly correlated with prognostic severity, with higher scores indicating a grimmer outlook (Fig. 11A). The nomogram's predictive prowess was reflected in a Concordance Index (C-index) of 0.866 (95% CI, 0.850–0.881), denoting substantial accuracy. Calibration curves further substantiated the nomogram's prognostic fidelity (Fig. 11B).

To strengthen the genuineness of the results, validated by adopting CGGA data, multivariate COX regression analyses have also delineated SLC3A2 alongside other clinicopathological indicators as salient prognostic constituents within glioma. Specifically, SLC3A2 (HR = 1.003, 95% CI [1.001–1.005], *P* = 0.005), IDH status (HR = 0.682, 95% CI [0.493–0.943], *P* = 0.021), 1p/19q codeletion (HR = 0.199, 95% CI [0.118–0.334], *P* = *P* < 0.001), and Age (HR = 1.015, 95% CI [1.002–1.028], *P* = 0.028) (Figure S3A-B).

Analogously, we devised a nomogram of the aforementioned independent prognostic determinants for OS. The robust predictive power of the nomogram was reflected by a C-index of 0.686 (95% CI 0.667–0.705) (Figure S3C). The rigor of the nomogram was verified with calibration curves further (Figure S3D).

**Figure 12:**
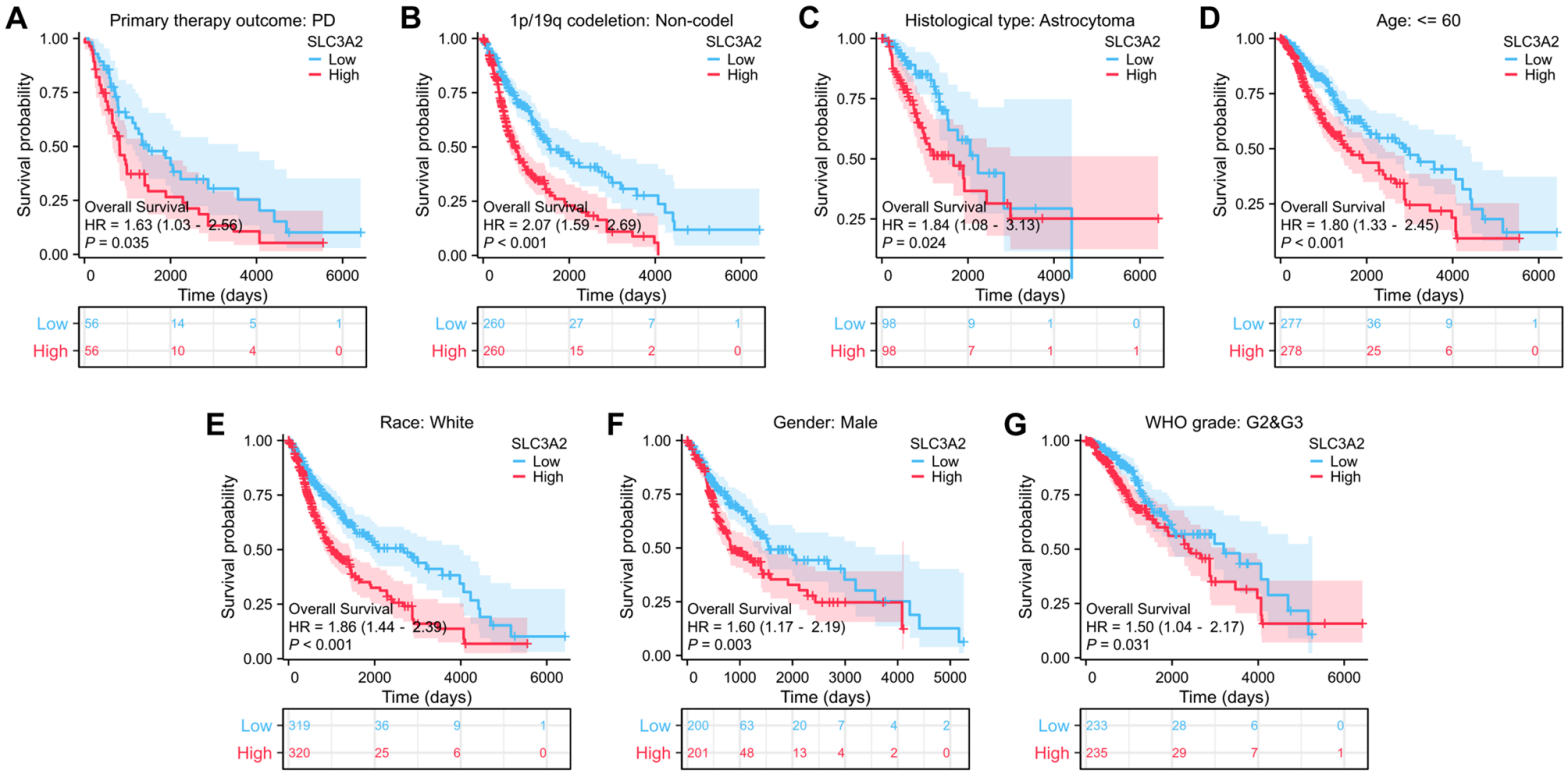
Prognostic relationship of SLC3A2 with clinical subgroups. (**A**–**G**) KM plots show the prognostic impact of SLC3A2^High^ and SLC3A2^Low^ in subgroups.

Subsequent investigations have highlighted the implications of SLC3A2 expression on diverse clinical subgroups, including primary therapy outcome, 1p/19q codeletion, histological type, age, race, gender, and WHO grade, all of which have shown that patients in the cohort with high SLC3A2 have a poorer prognosis (*P* < *P* < 0.05) (Figs. 12A-G).

### Functional insights from SLC3A2 knockdown in glioma pathobiology

**Figure 13:**
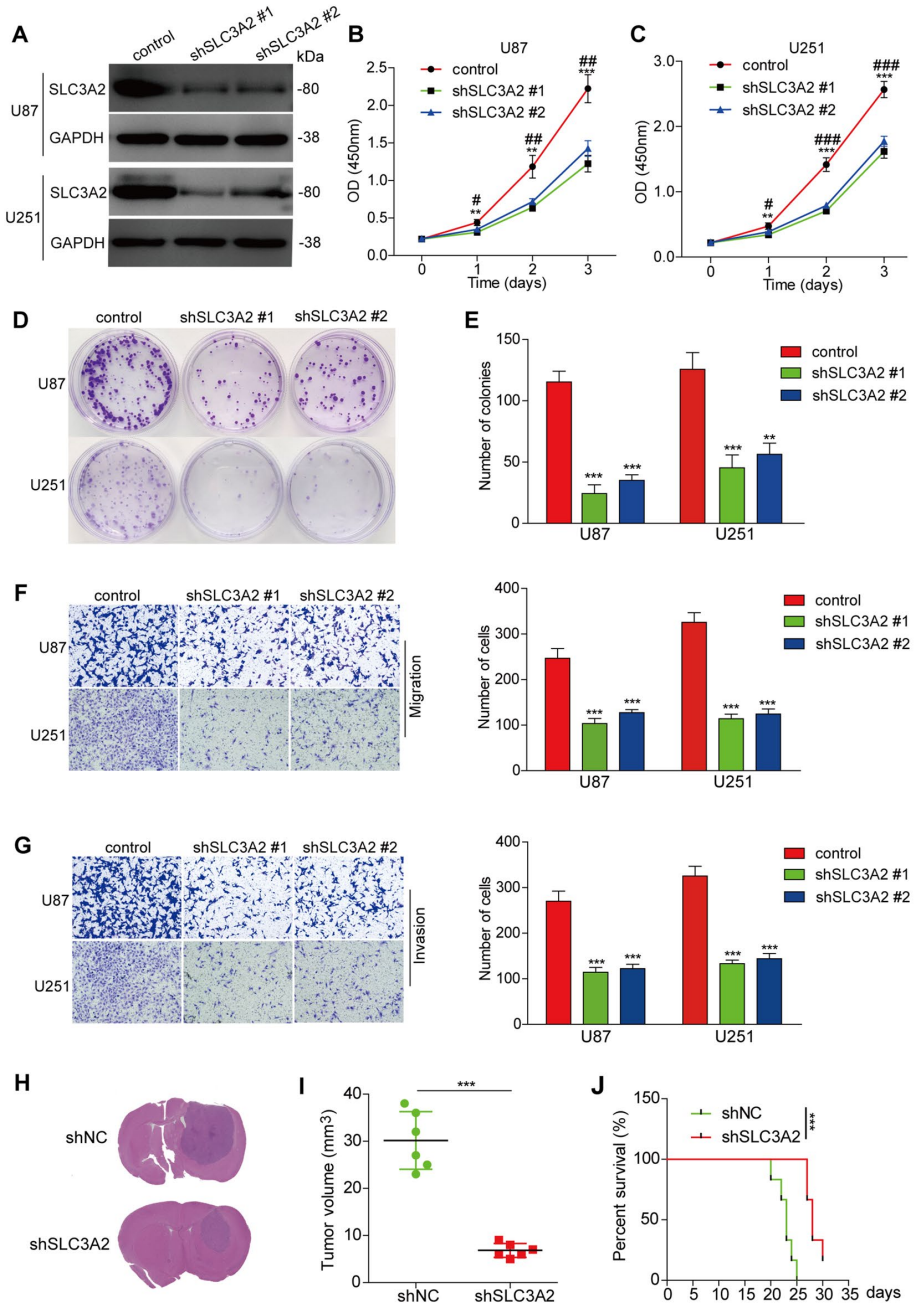
Knockdown of SLC3A2 attenuates the proliferation, invasion, and migration capabilities of glioma cells. (**A**) Implementing shRNA for SLC3A2 knockdown, we assess SLC3A2 protein levels via western blot analysis. (**B**–**C**) The proliferation of U87 and U251 cells, with and without the SLC3A2 suppression, is measured using the CCK-8 assay. The comparison between SLC3A2 shRNA #1 and the control, as well as SLC3A2 shRNA #2 against the control, showed statistical significance, with * *P* < 0.05, ** *P* < 0.01, *** *P* < 0.001; and # *P* < 0.05, ## *P* < 0.01, ### *P* < 0.001, respectively. (**D**–**E**) The colony formation test determines the colony-building ability in U87 and U251 cells post-SLC3A2 knockdown, revealing a significant difference when compared to the control shRNA. (**F**–**G**) Through the transwell assay, the migratory and invasive capacities of U87 and U251 cells, with or without SLC3A2 reduction, are evaluated. (**H**) Histological analyses of orthotopic glioma in mice from shNC and shSLC3A2 groups are presented. (**I**) The tumor sizes from orthotopic glioma xenografts are quantitatively analyzed. (**J**) Kaplan–Meier survival plots for mice with glioma highlight a significant improvement in survival rates for the shSLC3A2 group. * *P* < 0.05, ** *P* < 0.01, *** *P* < 0.001.

Moving past computational analysis, we embarked on in vitro experiments to investigate the role of SLC3A2 in glioma pathology. Intriguingly, SLC3A2 attenuation significantly curtailed proliferation, invasion, and migratory propensities in U87 and U251 glioma cell lines (Fig. 13A-G). Subsequently, the impact of SLC3A2 on glioblastoma was examined using a U87 orthotopic xenograft mouse model.

Findings indicated a substantial reduction in tumor sizes and an improvement in the survival rates of mice in the SLC3A2 knockdown group over 30 days, compared to the control group (Fig. 13H-J). The oncogenic nature of SLC3A2, demonstrated via both in vitro and in vivo studies, aligns with our bioinformatics findings, highlighting the critical function of SLC3A2 in the development of glioma.

## Discussion

Disulfidptosis, a newly identified type of cell death marked by the unusual buildup of disulfide bonds in cells like cysteine, plays a part in tumor metabolism and tumorigenesis, notably in gliomas^6,27^. Recently, research spearheaded by Gan Boyi et al. substantiates that elevated SLC7A11 expression predisposes cells to disulfidptosis, hinting at a potential functional role for disulfidptosis in glioma pathobiology. SLC3A2, a gene playing a role in disulfidptosis, is a subunit of the cystine/glutamate transporter regulating cysteine transport^8^. Elevated levels of SLC3A2 are linked to the emergence, metabolic activity, and advancement of several cancers, particularly lung, breast, squamous cell carcinoma of the head and neck, and liver cancers^10,11,28,29^. The specific function of SLC3A2 in gliomas, however, is not entirely clear. This work delves into the biological relevance of SLC3A2 in gliomas. Our results indicate a significant increase in SLC3A2 expression in tumor samples compared to normal ones, closely linking it to a poor prognosis in gliomas cases. Furthermore, our investigation has demonstrated that various immune markers and levels of immune infiltration in gliomas are correlated with the expression of SLC3A2. Additionally, Cox regression analyses suggest that SLC3A2 independently influences the prognosis of glioma. Experimental evidence also suggests that reducing SLC3A2 levels decreases tumor cell proliferation, migration, and invasion, and hampers glioma growth in vivo. Overall, these results suggest that SLC3A2 is an oncogene leading to poor outcomes in gliomas.

For a deeper understanding, pathway enrichment analyses were conducted on the identified DEGs. GO pathway analyses revealed significant enrichment in pathways closely related to sulfur and amino acid metabolism, including amino acid transport, sulfotransferase activity, and amino acid binding. Considering the disulfide bond-mediated cell death mechanism^6^, these pathways may be associated with the occurrence of disulfide bond-mediated cell death. This suggests that upregulation of SLC3A2 may induce disulfidptosis, thereby affecting glioma prognosis and metabolism. Moreover, KEGG pathway analysis highlighted the enrichment of MAPK signaling pathway, Rap1 signaling pathway, and HIF-1 signaling pathway, recognized as key pathways for tumor proliferation, development, and metastasis^30–32^. Through GSEA analysis, we further explored the functional characteristics of SLC3A2, discovering that its high expression inhibits the G-protein-coupled receptor signaling pathway. Notably, this pathway has a crucial function in maintaining the stability of the blood–brain barrier in the central nervous system^33^. Therefore, the inhibition of this pathway by SLC3A2 could lead to glioma hemorrhage, adversely affecting patient survival rates. In conclusion, SLC3A2 might promote tumor proliferation, progression, metastasis, hemorrhage, and metabolic changes through mechanisms like disulfidptosis, ultimately lowering the overall survival rate of glioma patients.

Genes functionally linked to SLC3A2 have been identified using circuits such as STRING. The PPI network has demonstrated several hub genes significantly involved with SLC3A2, such as SLC7A11, SLC1A5, and SLC7A5. These genes mediate various pathways such as disulfidptosis and autophagy, thereby regulating tumor progression^6,34^.

DNA methylation is a recognized mechanism involved in the aging process and cancer progression. Previous studies have shown that increased levels of promoter methylation led to significant reductions or even complete inhibition of gene expression^35^. Consistent with these established findings, our investigation further confirmed the association between low levels of SLC3A2-related methylation sites and increased SLC3A2 expression. Furthermore, we made an observation that GBM and LGG patients with higher levels of SLC3A2 promoter region methylation have a favorable prognosis.

The tumor microenvironment is characterized by its complex composition of various cells, molecules, and biochemical components that encapsulate tumor cells^36^. In this comprehensive survey, we meticulously studied the impact of the SLC3A2 gene on the tumor microenvironment from multiple perspectives. The presence of leukocytes in the tumor microenvironment has significant implications for tumor proliferation and metastasis. Previous studies have already indicated the presence of SLC3A2 expression in varied immune infiltrating cell populations, including CD8 T cells, mast cells, Treg cells, and dendritic cells^37–40^. Furthermore, utilizing analytical techniques such as CIBERSORT and ssGSEA, we unveiled a notable association between heightened SLC3A2 expression and enhanced infiltration of immune cells such as macrophages, NK cells, and neutrophils. Consequently, we took the logical next step and employed single-cell sequencing analysis to meticulously scrutinize each distinct cell cluster within the tumor microenvironment. This approach successfully demonstrated that SLC3A2 was found to be predominantly expressed in macrophages. The comprehensive immune cell infiltration analysis and single-cell sequencing results confirmed that SLC3A2 affected the proliferation of Macrophages. In this study, we further confirmed that increased M2 macrophages impacted the prognosis of SLC3A2^High^, and also found a significant positive correlation between SLC3A2 and M2 macrophage markers. Therefore, we concluded that SLC3A2 may be capable of influencing the tumor immune microenvironment of gliomas by regulating M2 macrophages.

Immune checkpoint blockade therapy is a critically important immunotherapy for cancer treatment, with common targets including anti-CTLA and anti-PD-1/PD-L1. Excessive activation of immune checkpoint pathways can suppress T cell activation, impair T cell immune function, and promote the proliferation of immunosuppressive cells, ultimately leading to tumor immune evasion^41,42^. This research identified a significant correlation between TIDE and SLC3A2 expression levels, indicating that elevated SLC3A2 expression may be predisposed to immune evasion, thereby diminishing the efficacy of immunotherapy. Furthermore, our analysis revealed a strong positive relationship between the levels of SLC3A2 expression and EMT scores. The EMT process serves as a crucial factor, modifying the tumor microenvironment via diverse pathways. Emerging research indicates that EMT not only facilitates tumor invasion and metastasis but also regulates the expression of immune checkpoints, thereby contributing to tumor drug resistance^21,43^. These insights underscore the multifaceted role of SLC3A2 in gliomas progression and the profound influence of SLC3A2 on the success of immune therapies and proposes its viability as a target for immunotherapy^44^.

In our investigation, bioinformatics analyses have indicated that elevated SLC3A2 expression not only has been associated with adverse clinicopathological features but also as a potential regulator of the tumor immune microenvironment, suggesting its involvement in modulating immune cellular dynamics, notably the M2 macrophage infiltration. Further, we have initiated an exploration into the role of SLC3A2 in tumorigenesis through a series of in vitro and in vivo experiments. These experiments provide preliminary insights into the gene's impact on cancer cell behavior and tumor growth.

In summary, in this study, we have identified SLC3A2 as a critical factor associated with poor prognosis and enhanced malignancy in gliomas. Nevertheless, there are limitations to our study: the experimental part of this study has not been extended to explore the mechanism of coordination between SLC3A2 and tumor immune infiltration. Understanding how SLC3A2 impacts the immune dynamics within the tumor microenvironment is crucial for our future studies. Such studies have the potential to uncover new therapeutic avenues for gliomas and other cancers in which SLC3A2 plays a key role, with important implications for the development of more effective therapeutic strategies.

## Conclusions

This study has successfully established the role of SLC3A2 in modulating immune infiltration and its impact on tumor proliferation, metastasis, and invasion. SLC3A2 potentially functions as an oncogene, altering the tumor microenvironment, thus playing a crucial role in the pathobiology of gliomas. This understanding underscores the importance of SLC3A2 as a novel prognostic marker and a potential therapeutic target, particularly in immunotherapy strategies, highlighting its significance in the broader context of cancer treatment.

### Supplementary Information

Supplementary Information 1.

Supplementary Information 2.

## Data Availability

The databases that serve as sources of research data are all publicly accessible. All of the original data used in this study is freely available at the websites or links provided in this article. Data relating to this investigation can be available from the corresponding author upon reasonable request. The UCSC XENA (https://xenabrowser.net/datapages/). The Cancer Genome Atlas (TCGA) (https://portal.gdc.cancer.gov/). Chinese Glioma Genome Atlas (CGGA). (http://www.cgga.org.cn). HPA database (https://www.proteinatlas.org/about). STRING database (https://string-db.org/). Molecular Signatures Database (MSigDB). (https://www.gsea-msigdb.org/gsea/msigdb/index.jsp). MethSurv (https://biit.cs.ut.ee/methsurv). TISCH2 (http://tisch.comp-genomics.org). TIDE (http://tide.dfci.harvard.edu/login/). TIMER 2.0 (http://timer.cistrome.org/).
