# Supplementary material for: Elevated SLC3A2 associated with poor prognosis and enhanced malignancy in gliomas
Source: Sci Rep. 2024 Jul 9;14:15758. doi: 10.1038/s41598-024-66484-1 (PMC11231275; doi:10.1038/s41598-024-66484-1)
Supplement: Supplementary file 1 — Supplementary Information 1. [file 41598_2024_66484_MOESM1_ESM.docx]

**Supplementary informations**

**Figure S1: Single-cell sequencing analysis results associated with SLC3A2.**

**Figure S2: Association between SLC3A2 expression and 10 common immune checkpoints (ICPs).**

**Figure S3: Validation analyses of independent prognostic variables.**

**Table S1: The gene set enrichment analysis (GSEA) results based on the expression levels of SLC3A2.**

**Table S2: Univariate and multivariate Cox regression analyses based on SLC3A2 expression and other clinicopathological variables.**


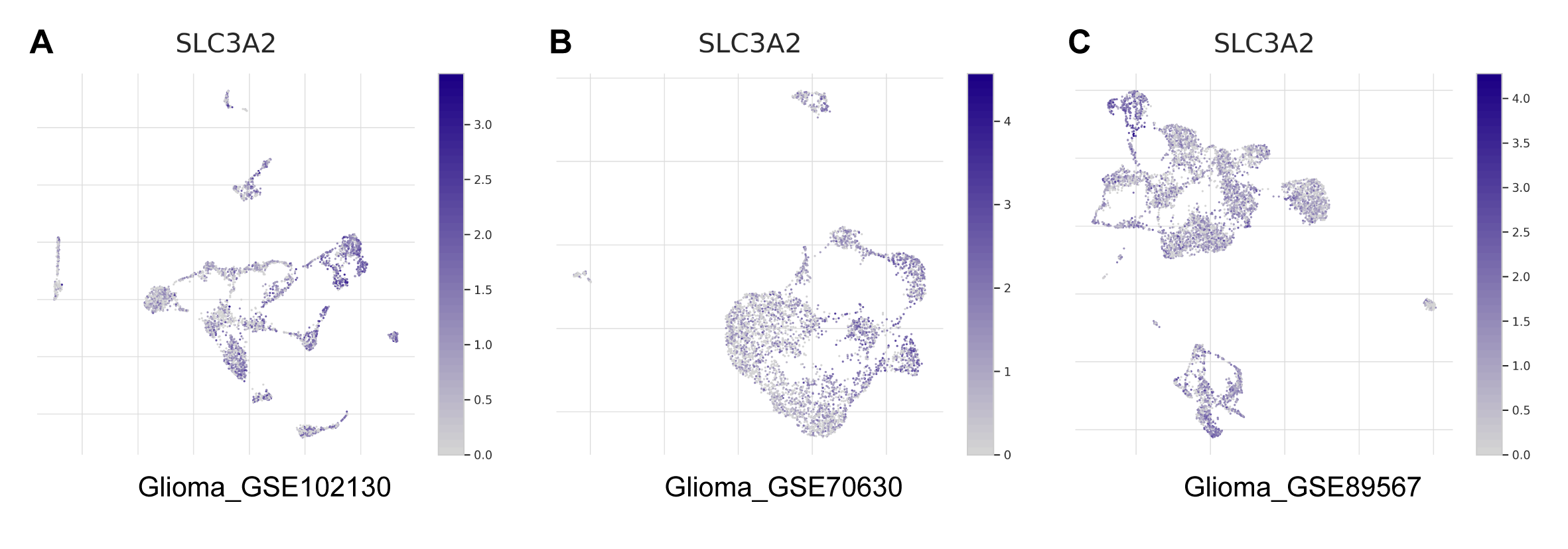


**Figure S1: Single-cell sequencing analysis results associated with SLC3A2.** (A-C) UMAP plots showing the expression levels of SLC3A2 in all cell clusters. Intensity of color (blue shading) corresponds to higher expression levels of SLC3A2 within the respective subgroups or individual cells.


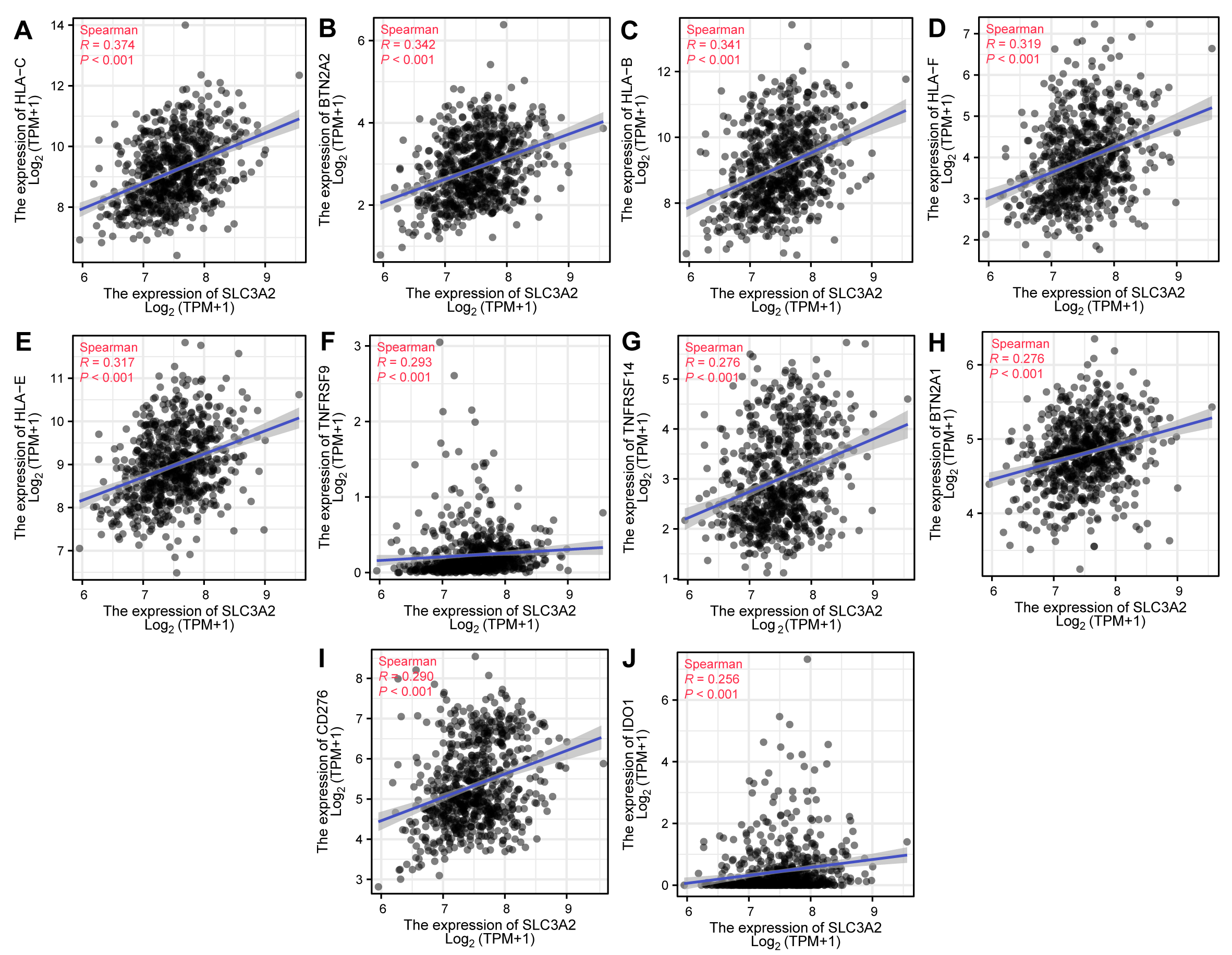


**Figure S2: Association between SLC3A2 expression and 10 common immune checkpoints (ICPs).**

**
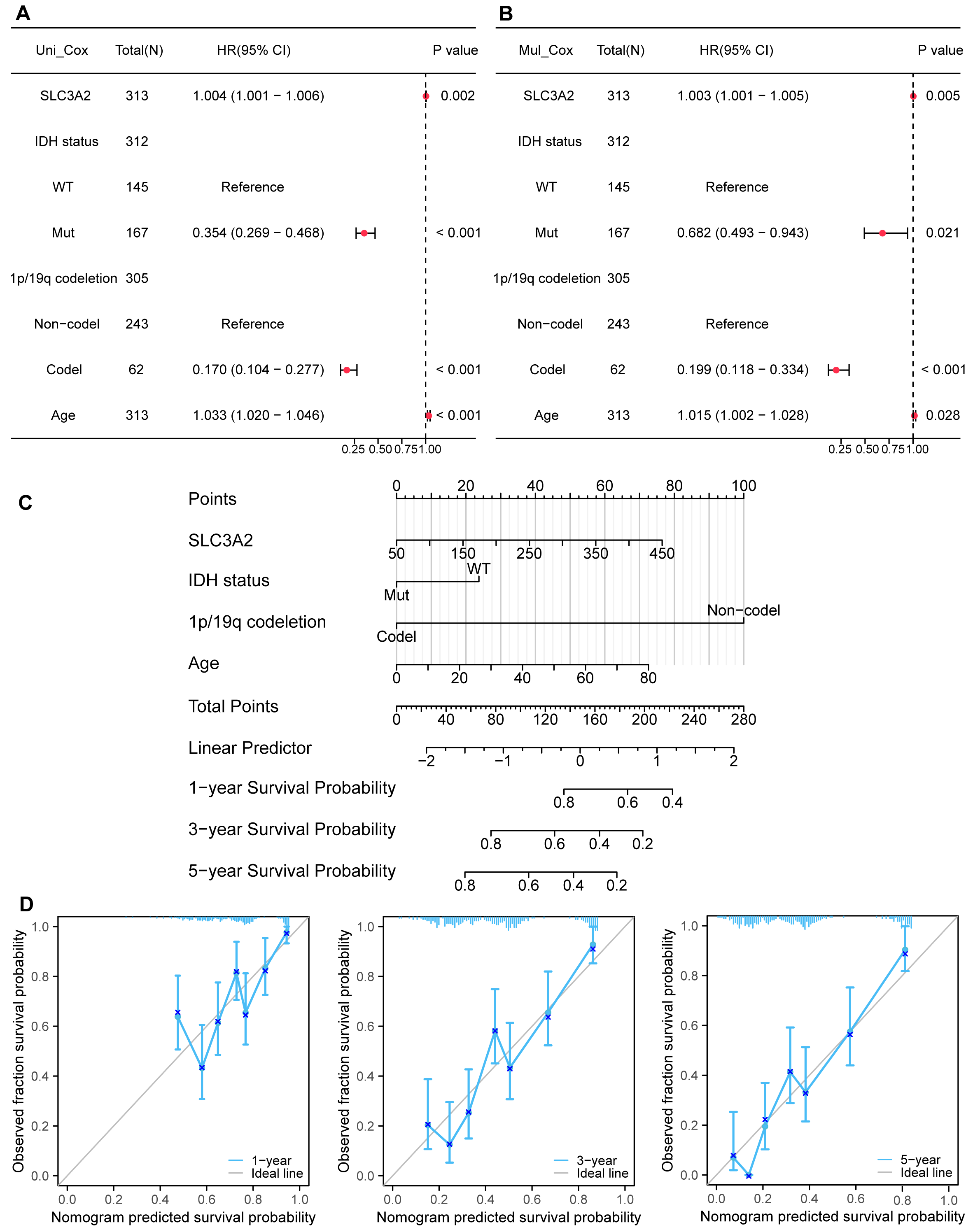
**

**Figure S3:** **Validation analyses of independent prognostic variables.** (A-B) Forest plots visualize the results of COX regression. (C) Nomogram of the prediction of overall survival. (D) Calibration curves for prognostic prediction nomograms.

**Table S1: The gene set enrichment analysis (GSEA) results based on the expression levels of SLC3A2.**

| **Gene set name** | **NES** |
| --- | --- |
| High expression |  |
| GO_NEGATIVE_REGULATION_OF_RNA_BIOSYNTHETIC_PROCESS | 2.920 |
| GO_NEGATIVE_REGULATION_OF_BIOSYNTHETIC_PROCESS | 2.787 |
| GO_PATTERN_SPECIFICATION_PROCESS | 2.592 |
| GO_REGIONALIZATION | 2.569 |
| GO_DNA_BINDING_TRANSCRIPTION_FACTOR_ACTIVITY | 2.467 |
| Low expression |  |
| GO_SYNAPSE | -2.175 |
| GO_G_PROTEIN_COUPLED_RECEPTOR_SIGNALING_PATHWAY | -2.257 |
| GO_SYNAPSE_PART | -2.274 |
| GO_VOLTAGE_GATED_CATION_CHANNEL_ACTIVITY | -2.410 |
| GO_NEURON_PART | -2.564 |
| KEGG_ Nicotine addiction | -1.937 |
| KEGG_ Neuroactive ligand-receptor interaction | -2.040 |
| KEGG_ GABAergic synapse | -2.148 |
| KEGG_ Serotonergic synapse | -2.168 |

**Table S2: Univariate and multivariate Cox regression analyses based on SLC3A2 expression and other clinicopathological variables.**

| Characteristics | Total(N) | Univariate analysis | |  | Multivariate analysis | |
| --- | --- | --- | --- | --- | --- | --- |
|  |  | Hazard ratio (95% CI) | P value |  | Hazard ratio (95% CI) | P value |
| SLC3A2 | 698 | 1.846 (1.445 - 2.358) | **< 0.001** |  | 1.630 (1.066 - 2.492) | **0.024** |
| WHO grade | 636 |  |  |  |  |  |
| G2&G3 | 468 | Reference |  |  | Reference |  |
| G4 | 168 | 9.538 (7.243 - 12.560) | **< 0.001** |  | 2.836 (0.962 - 8.364) | 0.059 |
| IDH status | 688 |  |  |  |  |  |
| WT | 246 | Reference |  |  | Reference |  |
| Mut | 442 | 0.116 (0.089 - 0.151) | **< 0.001** |  | 0.481 (0.293 - 0.792) | **0.004** |
| 1p/19q codeletion | 691 |  |  |  |  |  |
| Non-codel | 520 | Reference |  |  | Reference |  |
| Codel | 171 | 0.225 (0.147 - 0.346) | **< 0.001** |  | 0.448 (0.258 - 0.778) | **0.004** |
| Primary therapy outcome | 464 |  |  |  |  |  |
| PD | 112 | Reference |  |  | Reference |  |
| SD | 148 | 0.440 (0.294 - 0.658) | **< 0.001** |  | 0.475 (0.295 - 0.765) | **0.002** |
| PR | 65 | 0.167 (0.073 - 0.385) | **< 0.001** |  | 0.168 (0.060 - 0.467) | **< 0.001** |
| CR | 139 | 0.131 (0.063 - 0.273) | **< 0.001** |  | 0.243 (0.114 - 0.517) | **< 0.001** |
| Age | 698 | 1.066 (1.057 - 1.076) | **< 0.001** |  | 1.058 (1.041 - 1.074) | **< 0.001** |
| Gender | 698 |  |  |  |  |  |
| Female | 297 | Reference |  |  |  |  |
| Male | 401 | 1.250 (0.979 - 1.595) | 0.073 |  |  |  |
| Race | 685 |  |  |  |  |  |
| Asian | 13 | Reference |  |  |  |  |
| Black or African American | 33 | 1.578 (0.453 - 5.494) | 0.473 |  |  |  |
| White | 639 | 1.170 (0.374 - 3.657) | 0.787 |  |  |  |
