# Supplementary figures and images for: Elevated SLC3A2 associated with poor prognosis and enhanced malignancy in gliomas
Source: Sci Rep. 2024 Jul 9;14:15758. doi: 10.1038/s41598-024-66484-1 (PMC11231275; doi:10.1038/s41598-024-66484-1)

## Supplementary informations

### Whole uncropped images of the original Western blots

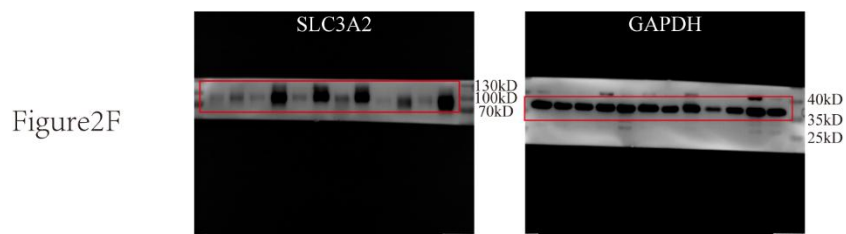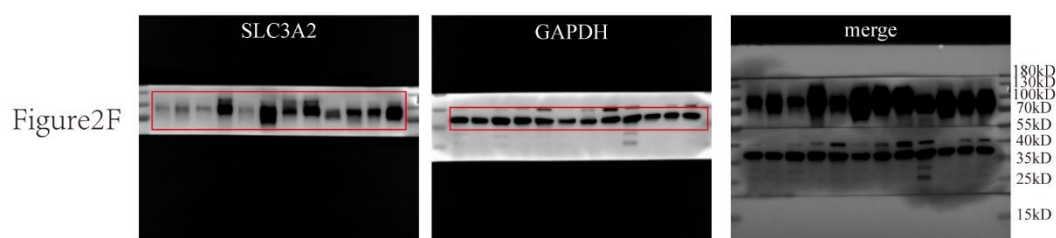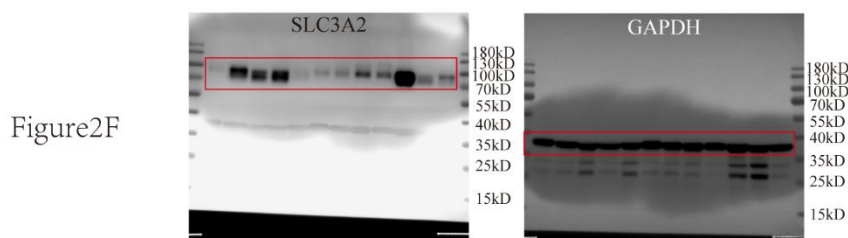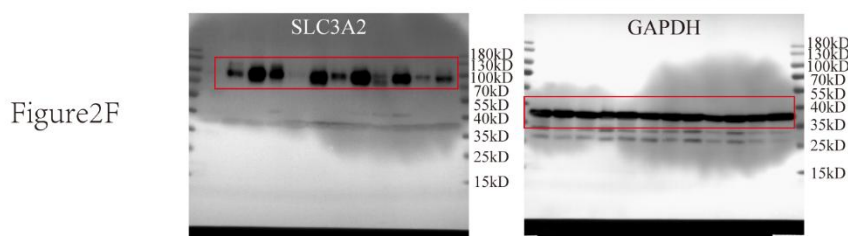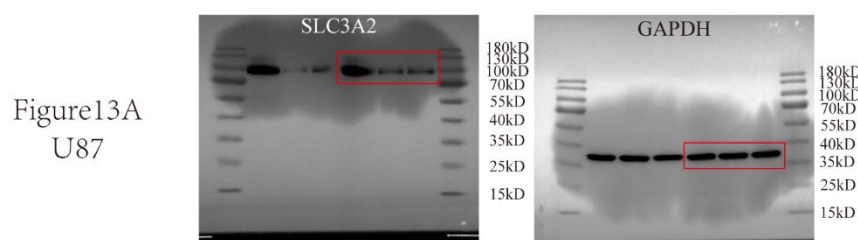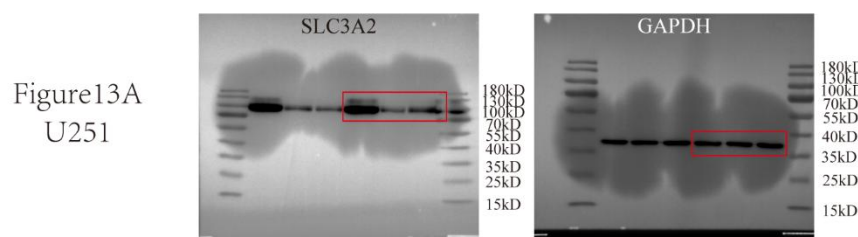

Supplement: Supplementary file 2 — Supplementary Information 2. [file 41598_2024_66484_MOESM2_ESM.pdf]
